# Spatially Extensive LFP Correlations Identify Slow-Wave Sleep in Marmoset Sensorimotor Cortex

**DOI:** 10.1523/ENEURO.0139-25.2025

**Published:** 2025-11-06

**Authors:** Paul L. Aparicio, Jeffrey D. Walker, Jason N. MacLean, Nicholas G. Hatsopoulos

**Affiliations:** ^1^Departments of Organismal Biology and Anatomy, Chicago, Illinois 60637; ^2^Neurobiology, University of Chicago, Chicago, Illinois 60637; ^3^Committee on Computational Neuroscience, University of Chicago, Chicago, Illinois 60637; ^4^University of Chicago Neuroscience Institute, Chicago, Illinois 60637

**Keywords:** marmoset, sensorimotor, sleep

## Abstract

Identifying neural signatures of slow-wave sleep (SWS) is important for a number of reasons including diagnosing potential sleep disorders and examining its role in memory consolidation ( Diekelmann and Born, 2010; Klinzing et al., 2019; Brodt et al., 2023). Studies of sleep in the common marmoset (*Callithrix jacchus*) have revealed similarities to humans and other nonhuman primates, including distinct sleep stages ( Crofts et al., 2001) and diurnal sleep patterns ( Hoffmann et al., 2012). Advances in applying wireless technology for recording neural activity during natural, unrestrained behaviors ( Walker et al., 2021) position the marmoset as an excellent model for studying sleep-related neural activity associated with learning. Here, we identify putative SWS epochs based on the spatially correlated activity of local field potentials (LFPs) recorded from a multielectrode planar array implanted in the sensorimotor cortex of two marmosets (one female and one male). The average correlation of the LFP signal measured between electrodes decreased gradually with the distance between pairs. We modeled this spatial structure as an exponential decay function, where the spatial decay constant varied significantly over time, reaching its lowest values during epochs where LFP power dynamics were consistent with SWS. These periods of widespread high correlations across the sensorimotor cortex closely matched SWS identification commonly used in rodent models based on the changes in power in the gamma (30–60 Hz) and delta/slow oscillation (0.1–4 Hz) frequency bands. These findings demonstrate that putative SWS epochs can be reliably identified using spatially correlated LFP activity across the sensorimotor cortex.

## Significance Statement

We demonstrate the reliable identification of putative SWS epochs based on correlated local field potential (LFP) activity recorded in the sensorimotor cortex of common marmosets during nighttime sleep. Slow-wave sleep, a distinct stage in the normal sleep cycle of humans and nonhuman primates, has been shown to play an important role in memory consolidation, and we demonstrate how spatial LFP correlations can provide an additional signature for SWS. Similar findings in other mammals suggest a broader relationship between sleep state and the spatial correlation structure across the sensorimotor cortex.

## Introduction

In mammals, birds, and even reptiles such as lizards (Shein-Idelson et al., 2016), sleep is essentially divided into repeating cycles, or periods, of rapid eye movement (REM) sleep and non-REM sleep. Neural activity during REM sleep is characterized by rapid, and desynchronized, low-amplitude neural activity not dissimilar to what is observed in awake recordings (Diekelmann and Born, 2010; Klinzing et al., 2019). Non-REM sleep, on the other hand, is characterized by numerous events that are observed in electric field recordings such as spindles, delta waves (4 Hz), and slow oscillations (<1 Hz; Steriade, 2003; Stickgold, 2005; Diekelmann and Born, 2010; Staresina, 2024). Based on the presence (or absence) of these events, non-REM sleep is further divided into periods of light and deep sleep. Periods of deep sleep are referred to as slow-wave sleep (SWS) due to the prominent presence of large-amplitude slow oscillations and significant increases in low-frequency power (0.1–4 Hz) in the electric field recording.

Slow oscillations (<1 Hz) are a prominent neural signature of SWS and have been observed to organize other sleep rhythms, such as spindle and delta (1–4 Hz) waves (Steriade, 2003), both of which have been linked to the acquisition of skilled motor behavior in rodents (Kim et al., 2019; Silversmith et al., 2020). Slow oscillations during SWS have also been shown to be related to cortical up and down states, where the probability of spiking activity is correspondingly increased or decreased (Destexhe et al., 1999). These periods of excitability and inhibition are thought to facilitate communication between the hippocampus and cortex through reactivation of cortical activity encoded during wakefulness. Reactivation is believed to have a prominent role in memory consolidation, allowing for transfer of new memories into long-term storage in the cortex (Diekelmann and Born, 2010; Brodt et al., 2023). These observations underscore how identifying further neural correlates of SWS periods during NREM sleep may further provide useful information for understanding memory consolidation.

The common marmoset (*Callithrix jacchus*) shares many advantages of rodent models for studying sleep, including being small, easily cared for, and well suited to laboratory settings (Abbott et al., 2003; Okano et al., 2012). Unlike rodents, marmosets and humans are diurnal (e.g., mostly awake during the day) exhibiting daytime wakefulness interrupted by naps and sleep states aligned with light/dark cycles (Crofts et al., 2001; Sri Kantha and Suzuki, 2006; Bukhtiyarova et al., 2022). Consequently, the marmoset is an excellent model for studying the neurophysiological basis of SWS, offering valuable insights into human sleep and the neural processes that underlie natural sleep. Furthermore, many of the experimental observations regarding the neural basis of sleep have been made in rodent and cat models, while relatively less work has been done in nonhuman primate models.

Here we examine the spatiotemporal coherence of local field potential (LFP) signals in the marmoset during nighttime sleep. Previous work (Destexhe et al., 1999) had suggested that a broad coherent spatial structure characterized NREM sleep across the parietal cortex in cats. We sought to extend this work in the sensorimotor cortex and, further, to examine the relationship between spatiotemporal structure and putative sleep state, as indicated by the dynamic changes typically observed in the LFP power spectrum during sleep. We observed extensive spatiotemporal coherence across the sensorimotor cortex during sleep in unrestrained marmosets. In comparison to previous work, these recordings were sampled at smaller spatial intervals (i.e., 400 μm) over a 4 × 4 mm patch of the sensorimotor cortex using a chronically implanted planar Utah array, thereby providing a more comprehensive examination of the spatiotemporal structure of the neural signal during SWS. Overall, our results demonstrate that broad spatiotemporal coherence in neural activity is a distinctive feature of the neural activity in epochs that demonstrate the temporal dynamics in LFP power consistent with SWS allowing for its identification. Furthermore, we show that the spatiotemporal coherence exhibited by the LFP signal across space is well correlated with the general dynamic interplay of the LFP power spectrum during nighttime sleep in the sensorimotor cortex.

## Materials and Methods

### Subjects

Two common (*Callithrix jacchus*) adult marmosets (subject TY, male and subject JL, female) were implanted with 96-channel planar Utah arrays (10 × 10 channel configuration, 1 mm long, with a 400 μm interelectrode distance, Blackrock Neurotech) at stereotaxic coordinates AP +9.8 and ML +4.5 targeting the forelimb area of the primary motor cortex (Burman et al., 2008; Paxinos et al., 2012). The subjects were pair housed in a 2 × 2 × 6 ft. enclosure furnished with perches, a nest box, and a hammock. The room was set on a 12 h on/off dark cycle and set to a temperature between 78 and 86°F with a target humidity of 30–70%. All surgical procedures and animal handling methods were performed in an AAALAC-accredited facility in accordance with the standards described in the Guide for the Care and Use of Laboratory Animals (National Research Council of the National Academies, 2011) and were approved by the University of Chicago Institutional Animal Care and Use Committee.

### Behavior

Subjects slept in a small hammock that was hung in their enclosure. The subjects typically entered the hammock before the room lights went out and left the hammock when the lights turned on the next morning. Sleep recordings were monitored through a standard IP camera. Recording sessions were started a few hours after the lights had turned off and lasted until the battery of the wireless recording headstage was depleted (∼1.5 h for TY). Further custom modifications allowed for longer sleep recordings in subject JL (3–5 h).

### Electrophysiology

**Figure 1.**
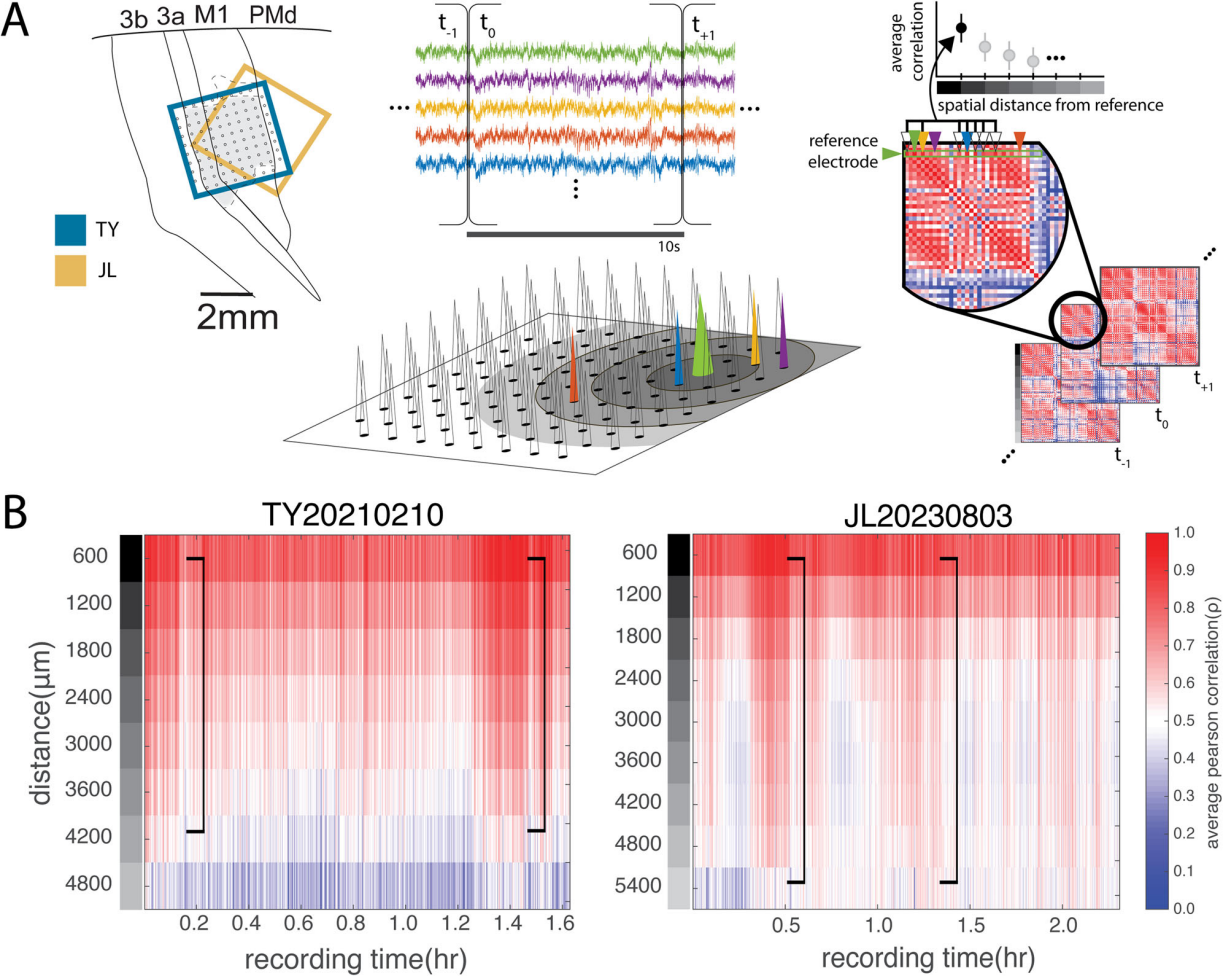
Correlation by distance functions demonstrate spatiotemporal structure during nighttime recordings (***A***, left panel). Recordings made from 96-channel Utah arrays chronically implanted in the sensorimotor cortex of two common marmosets, TY and JL. Schematic representation of the approximate location of the arrays in both animals determined by intracranial microsimulation (***A***, middle panel). The average pairwise correlation of the LFP as a function of distance was estimated over time. Nightly recordings were divided into 10 s epochs, and all the pairwise correlations between each electrode on the array were calculated for each epoch. For example, all the pairwise correlations between the electrodes in the darkest circles and central source electrode (green electrode) were averaged together for the 600 μm distance. Similarly, all the pairwise correlations between the electrodes in the next lighter colored ring and the source electrode were averaged together for the 1,200 μm distance. Note that each electrode will have different numbers of pairwise partners at each distance (***A***, right panel). ***B***, The average correlation by distance functions for each 10 s epoch over the course of a recording was generated by a weighted average across all the electrodes for example sessions in each subject. Broad spatiotemporal coherence is observed for brief periods during the recording in each subject (black brackets). See also Extended Data Figure 1-1.

Neural recording was conducted through a 96-channel Utah array using a wireless CerePlex Exilis headstage and a 128-channel Blackrock Cerebus system (Blackrock Neurotech). Neurophysiological signals were sampled continuously at 30 kHz and processed offline. The LFP signal was obtained by low-pass filtering the raw data at 250 Hz with a third-order Butterworth filter and downsampling to 1 kHz using a custom code written in MATLAB (MathWorks). We found the resulting signals often contained brief (<1 s) or longer (several seconds) wireless signal dropouts across all channels of the array. We treated these epochs as widespread correlated artifact and manually removed them from consideration in the analysis reported here using the FieldTrip toolbox in MATLAB (Oostenveld et al., 2011). A general statistical characterization of the signal dropout observed in our recordings is presented in Extended Data Figure 1-1. We used intracranial microstimulation and tactile and somatic receptive field mapping to verify that the array in TY covered forelimb M1 in addition to Areas 3a, 3b, and *a* portion of dorsal premotor cortex (PMd), while the array implanted in JL (at approximately the same location) covered more of PMd and some of 3a in addition to forelimb M1 (Fig. 1*A*, left panel). Array mapping sessions were conducted during quiet wakefulness in the evening prior to sleep.

### Distance correlations

A Pearson's correlation matrix was calculated on the *z*-scored signal from each electrode across the array for every independent 10 s interval in a recording. This produced a matrix containing all the pairwise correlations of the LFP signals during one 10 s epoch between each and every electrode pair. The pairwise distances between each and every electrode across the array were then estimated based on the geometry of the 10 × 10 design of the Blackrock Utah array with 400 μm interelectrode spacing. At each electrode (e.g., a source electrode), the pairwise correlation with every other electrode (e.g., each row of the correlation matrix) was Fisher *z*-transformed (Fisher, 1915), and the correlations were averaged according to the distance between the source electrode and the other electrodes in the pairwise correlations. This was done across independent bins of 600 μm distance (i.e., Bin 1 gathered all electrodes within 600 μm of the source electrode; Bin 2, all electrodes at distances >600 and <1,200 μm from the source electrode, etc.; Fig. 1*A*, middle panel). This produced a set of correlation by distance functions (one for each 10 s epoch during the recording) for each electrode on the array (Fig. 1*A*, right panel). The unique spatial position of each electrode and the geometry of the array resulted in different numbers of samples at some distances for different electrodes. To make a single set of correlation by distance functions to characterize the entire array for a single recording session, we took the average correlation across electrodes, weighting the value of a correlation at each distance on a particular electrode by the number of samples that contributed to that correlation at that distance. This produced a single correlation by distance function at each 10 s interval for a given recording session. Analytically derived 95% confidence intervals were used to identify correlation values at each electrode that were significantly greater than zero.

### Modeling distance functions

The correlation by distance function at each time point was Fisher-transformed and fit to a two-parameter exponential function by linear regression of the following form:

$$f(d)=Ae^{-d/\lambda },$$

where *A* is the initial value parameter and *λ* is the spatial decay constant or the distance across space that governs the decay rate. In a standard exponential decay function, the initial amount before the decay begins would logically be 1. At a distance of zero, we would expect the correlation function to also be 1, making the standard exponential decay function a rational choice to model the way that pairwise correlations decay across the cortical surface, as was done by Steriade and colleagues (Destexhe et al., 1999). However, in this study the correlation function is Fisher *z*-transformed so as to make the values normally distributed with a stable variance as would be appropriate for performing a linear regression on the Pearson correlation as a function of distance. This transform means that the initial value would no longer be 1; therefore, we allowed the initial value to be fit as a free parameter by the data. Additionally, we employed a power law model to describe our data of the following form:

$$f(d)=A/d^{-b},$$

where *A* is the initial value and *b* is the power value governing the spatial decay. Finally, we characterized the distribution of parameters we obtained at each time point for a recording session by fitting a line to the 2D scatter of the parameters with a Type 2 regression, where both parameters have variance (Draper and Smith, 1998).

### Identification of SWS

We used methods similar to those described in the literature for identifying putative SWS epochs in rodents using the well-known dynamics in LFP power during sleep (Kim et al., 2019; Silversmith et al., 2020). Briefly, we discarded data from channels that were broken or contained frequent readily visible signal artifacts. We then *z*-scored each of the remaining channels and averaged across all channels to create a single “virtual” LFP signal (vLFP) representing the entire array. The power spectrum of this vLFP signal was estimated using the Chronux toolbox (Mitra and Bokil, 2007; chronux.org) within nonoverlapping 10 s signal epochs. A slow oscillation (SO)/delta band signal was created by averaging the power from 0.1 to 4 Hz in each epoch. Similarly, a gamma band signal was created by averaging the power for frequencies within 30–60 Hz. Sleep states were identified using a Gaussian mixture model classifier with three clusters (though similar results were obtained with a *k*-means classifier) to identify periods where (1) SO/delta band power was greater than the gamma band signal power, (2) the gamma band signal power was greater than the SO/delta band signal power, and (3) time points where neither of (1) nor (2) was dominant (i.e., the null category). In other words, the null category indicated that neither the gamma nor the delta band exhibited modulation that was classified as either of the two other categories. Here we classify SWS as the cluster where SO/delta power is greater than gamma power, putative REM/awake epochs as the cluster where gamma power is greater than SO/delta power, and a null state where both SO/delta and gamma band power were approximately equivalent. These characterizations were validated through analysis of the head movement extracted from the video of an independent sleep recording session (Extended Data Fig. 3-3).

### Code accessibility

All calculations and analysis were carried out with custom scripts written in MATLAB (MathWorks) and are available at https://github.com/hatsopoulos-lab/Spatial-correlations. The video head tracking was performed with the progressive tracking software package in Python (Mielke et al., 2020).

## Results

We recorded neural activity wirelessly over multiple nights, while marmosets slept in their regular sleeping hammock. We report here the neural activity collected during four noncontiguous recording sessions in subject TY, and two separate, noncontiguous recording sessions in subject JL. All recordings took place in the subjects’ home enclosure. The broadband (<250 Hz) LFP was used to measure the pairwise correlations across the sensorimotor cortex, covering parts of Areas M1, Areas 3a and 3b in the somatosensory cortex, and PMd in two marmoset subjects (TY and JL).

### Correlation by distance functions reveal widespread, dynamic spatial structure in LFPs

We estimated the pairwise Pearson's correlations between the *z*-scored broadband LFP signal recorded on each of the 96 electrodes on the array for nonoverlapping 10 s epochs during the entire recording session (Fig. 1*A*, right panel). We employed relatively long temporal epochs to describe the correlation structure, but qualitatively similar results were observed for the fit distributions when using shorter time intervals (6 s or 1 s; Extended Data Fig. 2-1). Pairwise correlations between the electrodes across the spatial extent of the array were pooled together as a function of distance by averaging the Fisher's *z*-transform (Fisher, 1915) of the pairwise correlations for electrodes within a given distance interval from a particular source electrode (Fig. 1*A*, middle panel). Therefore, a given pairwise correlation contributed uniquely to the estimated correlation by distance function for a given source electrode during a particular time epoch. The results were then averaged across source electrodes. For the data presented here, we used a distance interval of 600 μm, but similar results were observed for a distance interval of 400 μm (data not shown).

10.1523/ENEURO.0139-25.2025.f1-1 Figure 1-1 **Characterization of Wireless Signal Dropout During Sleep Recordings. (A)** Duration of wireless signal dropouts: Histograms depict the duration of dropouts observed during all recording sessions. Fewer instances of signal dropout were observed in subject TY’s recordings, which were generally fewer and of longer duration than observed with subject JL. All x-ranges identical in main axes of **(A).** Insets show the distribution for the longer duration dropouts in subject TY’s recordings. Red lines depict the mean of the distribution. **(B)** Frequency of Dropouts: Histograms depict the number of 10 second epochs containing the number of dropouts listed on the x-axis. Note that there were no dropouts observed in session TY20210210, so all the epochs had zero dropouts. **(C)** Intersample interval for the dropout distribution in each recording. In all sessions, most of the dropouts happened in quick succession (the bin closest to zero is the largest). Download Figure 1-1, TIF file.

The average (across electrodes) correlation by distance functions across time for a single example sleep recording in each subject demonstrates several prominent features (Fig. 1*B*). Not surprisingly, correlations appear to be stronger on average for spatial locations closer to the source electrode (e.g., at smaller distances) gradually decaying over space. Additionally, the average pairwise correlation remains significantly elevated above zero at the furthest distances measurable across the recording array (∼5.5 mm), seemingly reaching a plateau (*ρ* ∼ 0.5). This spatially extensive static modulation (e.g., the plateau) was most reflected by power in the delta (1–4 Hz) band (Extended Data Fig. 2-2). Another apparent feature is that the correlation across distances is dynamic rather than static over time. Furthermore, there is a pronounced and sustained elevation of the correlation across space for some epochs of the recording (Fig. 1*B*, brackets). A final observation of note is that correlations at the two furthest distances appear considerably more variable than at all other distances. This is due to the geometrical arrangement of the probes on the array. The number of electrode pairs at these distances was small and variable, resulting in highly variable estimates.

### Exponential decay adequately describes spatial correlations

**Figure 2.**
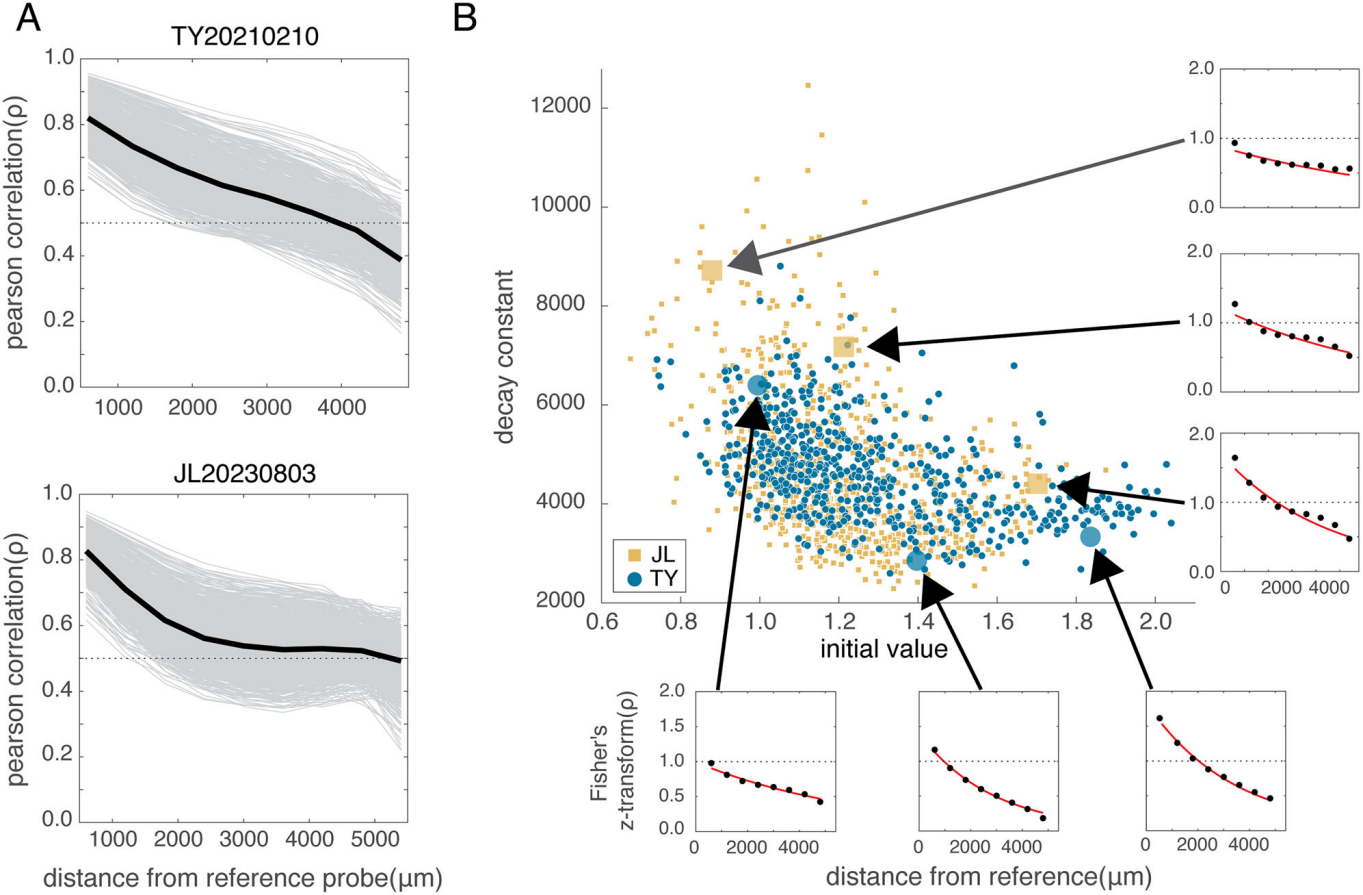
Spatiotemporal structure in the LFP signal during sleep can be modeled by an exponential decay function. ***A***, The correlation by distance averaged across electrodes during an example recording session in each subject. Each gray line shows the average pairwise correlation across distance in an independent 10 s epoch during the recording, while the black line shows the mean. ***B***, Each time point in the example sessions in ***A*** was variance normalized using Fisher's *z*-transformation and fit to an exponential function with two parameters (the initial value and spatial decay constant; see Materials and Methods). The scatter of these parameters is depicted for the example recording in each subject (TY, blue circles; JL, yellow squares). The parameters were similarly correlated in all recording sessions (four sessions in TY and two sessions in JL). Example points from the parameter distributions in each subject are plotted in the breakout plots and depict the change in the fits along the correlated axis. At low initial values, the decay constant tended to be higher and the correlation by distance functions changed slowly over space. Higher initial values resulted in a more aggressive decay in the function. See also Extended Data Figures 2-1 and 2-2.

We sought to quantify these observations across time by fitting an exponential decay model to the correlation by distance function at each time point. The exponential decay function is an intuitive approach to modeling the gradual decay in the pairwise correlations between electrodes across cortical space and performed marginally better than a linear model and at least as well as a Gaussian model with more parameters, though a power law model also fit our data well (Extended Data Figs. 3-1, 4-2). In both subjects, the average pairwise Pearson's correlation varied from *ρ* = ∼0.8 at the shortest distance interval (*D* = 0–600 μm) to *ρ* = ∼0.5 at the furthest reliable distance interval (*D* = 4,200 μm; Fig. 2*A*; see Extended Data Fig. 4-1 for similar plots in all recorded sessions). The values were similar across all recorded sessions ( $TY_{\bar{D}(600)}=0.82$; $CI_{95\text{\%}}[0.81,0.84]$; $TY_{\bar{D}(4200)}=0.49$; $CI_{95\text{\%}}[0.48,0.50]$; $JL_{\bar{D}(600)}=0.830$; $CI_{95\text{\%}}[0.826,0.835]$; $JL_{\bar{D}(4200)}=0.526$; $CI_{95\text{\%}}[0.519,0.533]$). To fit the correlation by distance functions by linear regression to an exponential decay process (see Materials and Methods), the correlations at each time point were Fisher *z*-transformed. The resulting fit had two model parameters: an initial value (the value of the Fisher *z*-transformed correlation at a zero distance, prior to any decay) and a decay constant that describes the distance over which the correlation values decrease by an amount proportional to the starting amount. As described earlier, distances greater than 4,200 μm often had very few observations making the average correlation at these distances unreliable. To account for this, we used a weighted least squares regression to find the optimal parameters for the model, where the weight at each distance was proportional to the number of observations that made up the correlation value at a given distance. Similar fits were observed when we excluded correlation values at distances that contained fewer than 20% of the average number of observations seen on other electrodes.

10.1523/ENEURO.0139-25.2025.f2-1 Figure 2-1 **Pairwise distance correlations estimated using different timescales. (A)** The average, across electrode, pairwise correlation as a function of distance across the array is plotted for each time point during the four recording sessions in TY and two recording sessions in JL. The lilac traces depict the results when a timescale of 1 second was used to estimate the pairwise correlations, while the lighter gray represents the same data estimated on a 6 second timescale. The thicker line of similar hue represents the mean. Shorter timescales produce a larger variance in the distribution of the correlation over space, and result in lower correlations on average: the mean of the distance functions for the 1 second timescale (heavy grey lines in **A**) is lower than it is for the 6 second timescale (heavy blue lines in **A**). **(B,C)**. Scatter plots for the exponential model parameters fit to the data in each recording session shown in **(A)** for the 1 second and 6 second time scales are showing the same qualitative trend as the broadband data: the spatial decay constant (λ) estimated from the data is anticorrelated with fitted initial value parameter. Download Figure 2-1, TIF file.

10.1523/ENEURO.0139-25.2025.f2-2 Figure 2-2 **Average pairwise correlation over distance in narrowband frequencies.** The narrowband power in the delta (1-4 Hz), beta (16-24 Hz), low gamma (25-55 Hz), and high gamma (65-140 Hz) frequencies were analytically derived from the broadband LFP signal obtained during nighttime sleep recordings in two marmoset subjects (TY and JL). Each plot depicts the average (across electrodes) pairwise correlation over the spatial extent of an array located in the forelimb representation of sensorimotor cortex (gray lines). The average over time is depicted as the black line in each plot. Spatially extensive high correlations are observed dominantly in the delta band across both subjects and all sessions. The peak correlation, in addition to the overall correlation, decreases in higher frequency bands. Download Figure 2-2, TIF file.

The fit parameter distributions for an example recording session in each subject are depicted by relating the spatial decay constant to the initial value, fit at each time point (Fig. 2*B*). Each point in the plot is an independent 10 s period during a different recording session in each subject (blue circles for subject TY and yellow squares for subject JL) obtained on different days. In these example recording sessions, there is an obvious anticorrelation between the two parameters: when the initial value is low, the decay constant tends to be higher and vice versa. This is demonstrated in the breakout plots, where the Fisher-transformed correlation by distance functions, for example, time points which are characterized by relatively low, medium, or high initial values, are plotted along with the corresponding exponential fit (shown in red) for each subject. The examples indicate that during certain periods of the nighttime recording, the average Fisher-transformed pairwise correlation of the LFP signal was significantly elevated across all distances and exhibited increases at distances near zero. We quantified the linear relationship between the two-parameter estimates from the exponential fit by performing a Type 2 linear regression to the parameter estimates, thereby accounting for the variance in both the initial value and decay constant over time. The average slope of the linear fit across all recorded sessions in each subject (TY, $\bar{m}=-3,958$; $m_{\mathrm{sd}}=851$; JL, $\bar{m}=-6,900$; $m_{\mathrm{sd}}=669$) was significantly different from zero (TY_(95%CI)_ = [−4,432, −2,764]; JL_(95%CI)_ = [−7,827, −5,973]; bootstrap-derived 95% confidence interval on the slope of the linear fit). This effect was present in each recording session for both subjects.

### Spatial structure in LFP correlations indicates sleep state

**Figure 3.**
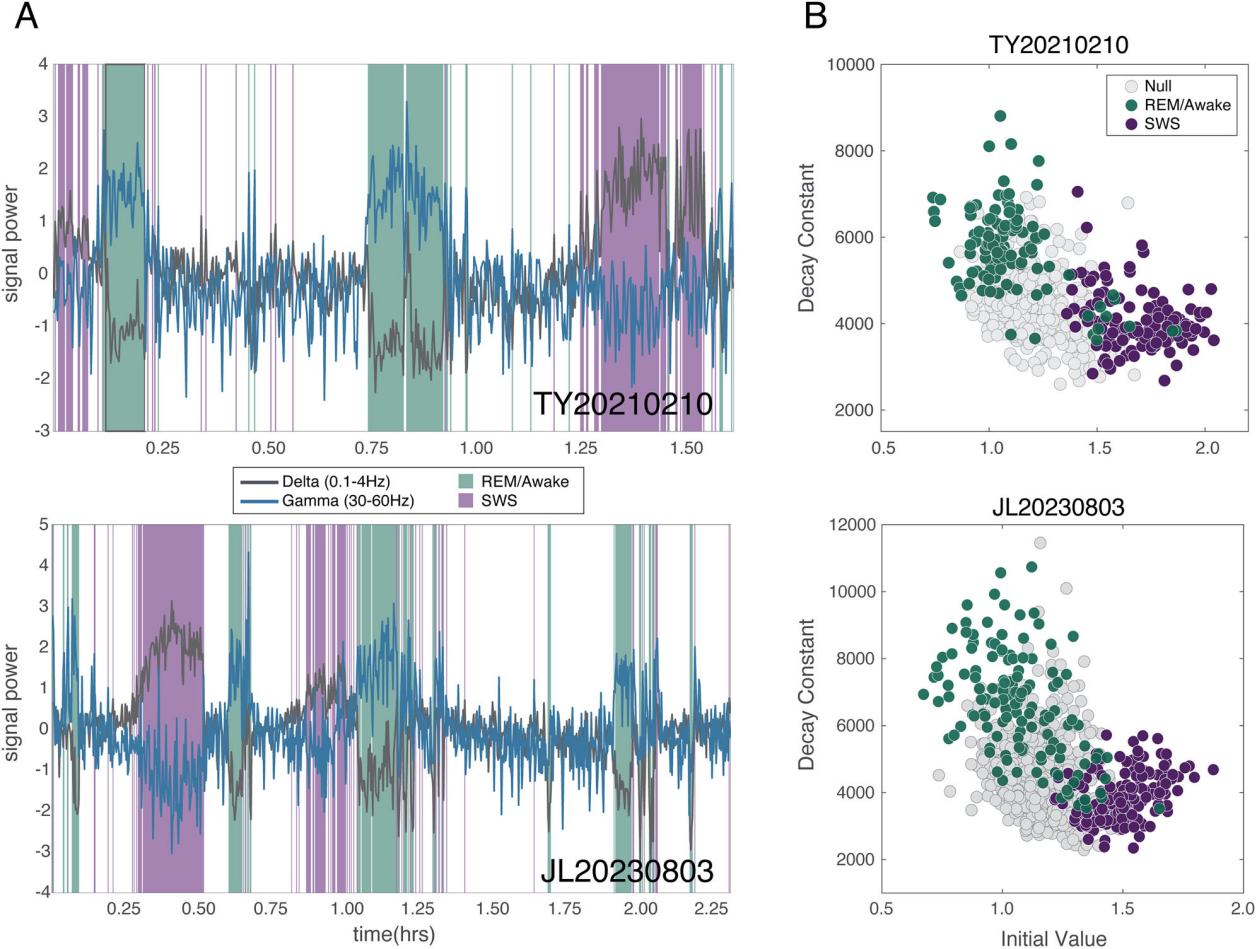
Sleep states display characteristic parameter values of exponential decay models. ***A***, Each time point in the example sessions for each subject were assigned a state determined by clustering the average power in the SO/delta (0.1–4 Hz) and gamma (30–60 Hz) band estimated at each time point (10 s epochs). The clustering largely reflects the observation that delta band power increases while gamma band power decreases during SWS. Similarly, REM/awake states are characterized by increases in gamma band power with decreases in SO/delta band power. ***B***, The states assigned to each time point were applied to the fit parameters described in Figure 2. Time points that were labeled as SWS by delta and gamma power dynamics tended to be characterized by lower decay constants and higher initial values. See also Extended Data Figures 3-1–3-3.

As the distribution of the parameter fits varied continuously, we sought to examine how this spatiotemporal structure coincided with the more traditional sleep classification used in sleep research. We performed a sleep classification based on only the LFP signal, taking advantage of the observation that SWS is characterized by increases in the delta (1–4 Hz) and slow (0.1–1 Hz) band power with concomitant decreases in the gamma (30–60 Hz) band power (Kim et al., 2019). We bandpass filtered the broadband LFP signal into SO/delta (0.1–4 Hz) and gamma (30–60 Hz) band signals and observed clear epochs of delta and gamma band power increases that are indicative of the classic pattern of alternating SWS and REM epochs (Fig. 3*A*) previously observed across many sleep studies including those in marmosets (Crofts et al., 2001; Bukhtiyarova et al., 2022).

10.1523/ENEURO.0139-25.2025.f3-1 Figure 3-1 **Fit error for models of the average pairwise correlation as a function of distance.** Linear, exponential, gaussian and power law models were fit to the average pairwise correlation across space in both subjects. The smoothed probability density estimates for each distribution of the mean squared error (MSE) is plotted for each model. Density estimates were carried out on the empirically observed distribution of the residuals fit for each model using Matlab’s ksdensity function (Mathworks, Natick, MA). A vertical line notes the mean of each distribution. The distribution of the MSE for the power law decay model tended to peak at lower values in subject TY, but comparable to the Gaussian model in subject JL. In subject TY, the distribution for exponential model is comparable to the performance of the Gaussian model across all the recording sessions. In subject JL, the error from the exponential model is broad and distributed at a higher mean than the Gaussian model. Linear models provide the highest error in both subjects. Download Figure 3-1, TIF file.

10.1523/ENEURO.0139-25.2025.f3-2 Figure 3-2 **Simple Threshold Model Significantly Predicts Putative SWS Epochs.** We tested a model generated by thresholding the initial value parameter fitted to each time point in a session in predicting SWS epochs on each recorded session. The fitted parameter was also smoothed in time. The threshold and smoothing value were obtained by minimizing the resulting error of the prediction on all the other sessions in a leave-one-out training paradigm. **(A)** The error distributions from shuffling the SWS labels show that the model prediction error (listed below each distribution) for each session was well below the 95% confidence interval of the shuffle distribution. **(B)** Estimates of accuracy (the proportion of hits and correct rejections) and precision (the proportion of hits to the sum of hits and false alarms) for each session. One session had a relatively low precision as shown in the plot. This session was also the one that had the least number of SWS epochs. Overall, this very simple model did reasonably well in predicting which epochs had LFP dynamics consistent with SWS. Similar results were found using the same threshold/smoothing model with the decay constant or the ratio of decay constant to initial value as the parameter to threshold (not shown). Download Figure 3-2, TIF file.

10.1523/ENEURO.0139-25.2025.f3-3 Figure 3-3 **Video Analysis of Movement During Sleep.** Video of sleep session was used for validating of sleep scoring. Note the neural recording headstage and charging cable indicating the subject of recording. Subject movement was quantified by tracking the movement of the subject’s head in each videoframe using the progressive tracking (Mielke et al., 2020). The x-y pixel-wise position of a point on the recording headstage was converted to head speed over the entire course of the recording, and sleep staging was conducted using the same 3 state (SWS, REM/Awake, and Null) model reported in the paper. **(A)** The average speed in each state over the entire recording shows that most movement occurred during REM/Awake, with a non-significant trend of movement in the Null state being larger than that during SWS. **(B)** Video frames depicting movement during example time points displayed in **(C)**. The sleep state is shown on the colored background, with log (head speed) plotted on the upper plot in blue and delta (0.1-4 Hz) and gamma (30-60 Hz) power plotted in black and gray on the bottom plot. A significant amount of movement is observed in the last REM/Awake epoch, where the animal is clearly shown awake. The Null epochs contain both periods of movement and no movement, as depicted in the first two video example frames, and likely represent transition states between REM and NREM states, lighter NREM states, and noise. Download Figure 3-3, TIF file.

Utilizing unsupervised clustering (Gaussian mixture model, though similar results were observed using *k*-means, data not shown) to cluster each time point with the SO/delta and gamma band signals as features enabled us to classify each 10 s epoch in the data into one of three states (SWS, REM/awake, and neither). These classifications were then applied to the scatter of fit parameters (Fig. 3*B*, same as Fig. 2, but with the sleep states of specific time points, determined from the clustering, depicted in color: green, awake/REM; purple, SWS; gray, neither). In both subjects, time points classified as SWS epochs tended to cluster at lower spatial decay constants. The average spatial decay constant and its standard deviation during SWS epochs across all recording sessions for subject TY ( $TY_{\bar{\lambda }}\;=4,128$; $TY_{\lambda_{\sigma }}=697$) and for subject JL ( $JL_{\bar{\lambda }}\;=3,852$; $JL_{\lambda_{\sigma }}=673$) were lower than the average spatial decay constants observed during awake/REM epochs, which tended to have higher spatial decay constants on average (subject TY, $TY_{\bar{\lambda }}\;=5,798$; $TY_{\lambda_{\sigma }}=1,238$; subject JL, $JL_{\bar{\lambda }}\;=6,482$; $JL_{\lambda_{\sigma }}=1,472$). The null state had spatial decay values distributed between the other two states, where the average spatial decay constant in the null epochs for subject TY ( $TY_{\bar{\lambda }}\;=4,532$; $TY_{\lambda_{\sigma }}=844$) and subject JL ( $JL_{\bar{\lambda }}\;=4,232$; $JL_{\lambda_{\sigma }}=1,184$). Time periods classified as SWS epochs, based on exponential fit parameters, well characterized SWS epochs as identified by traditional patterns in delta and gamma band power (Extended Data Fig. 3-2).

### Sleep state highly correlated with spatial structure of LFP

**Figure 4.**
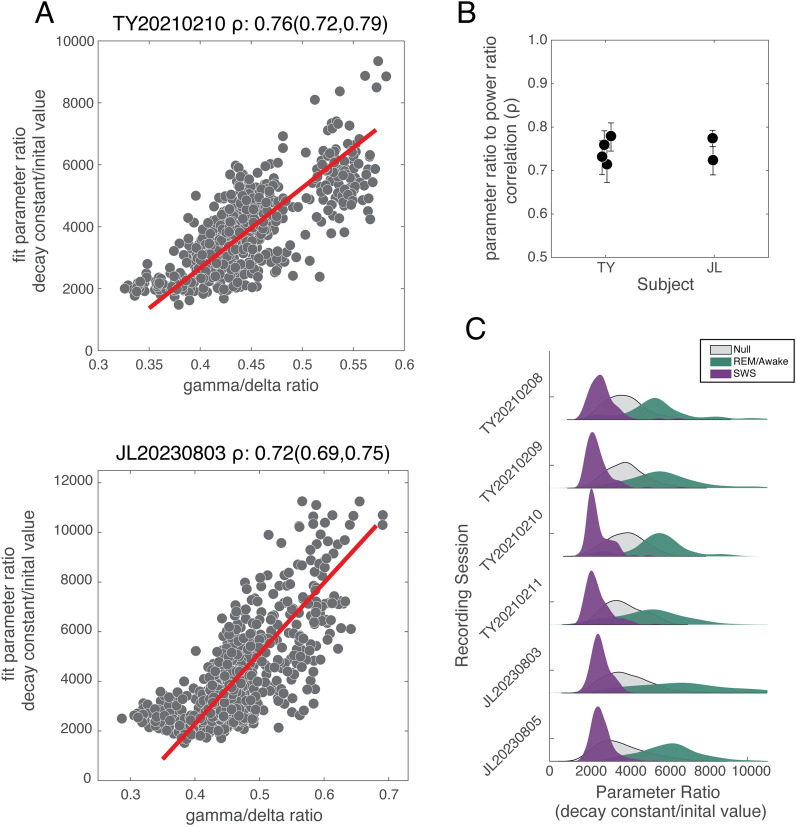
Sleep states are correlated with exponential decay model parameter values. ***A***, The decay constant estimated at each time point was normalized by its initial value (e.g., the ratio of the fit parameters estimated for each time point) and was highly correlated with the ratio of the narrowband power between the SO/delta (0.1–4 Hz) and gamma (30–60 Hz) bands. The SO/delta and gamma band power dynamics are largely reflected by SWS and awake/REM in the LFP signal. Pearson's correlation between the normalized decay constant and the ratio of delta to gamma band power is described in the title for each example session along with its 95% confidence interval. ***B***, Pearson's correlation between the two ratios was similar in value and significance for all recorded sessions studied. ***C***, The putative sleep state labels for each time point (described in Fig. 3) were applied to the normalized decay constant estimated at each time point. Probability density functions were then estimated for each sleep state category. Time points labeled SWS (purple) and REM/awake (green) were widely separated with putative SWS having lower normalized decay constants compared with REM/awake. See also Extended Data Figures 4-1 and 4-2.

The apparent correlation of our estimated sleep state to the parameter distribution suggests that there may be a direct relationship between sleep state and the spatial structure of the average pairwise correlations. To examine this idea, we looked at how the ratio of the fit parameters compared with the ratio of the gamma/delta narrow band power. We reasoned that the ratio of delta/SO (0.1–4 Hz) to gamma (30–60 Hz) band power should be smaller during putative REM/awake and larger during putative SWS. We would also expect this to be true for the ratio of the decay constant to the initial value estimated from fitting each time point to an exponential function. There is a clear linear relationship between the two ratios demonstrated by a significant positive Pearson's correlation (Fig. 4*A*; TY, *ρ* = 0.76 [0.72, 0.79]_95% CI_; JL, *ρ* = 0.72 [0.69, 0.75]_95% CI_; analytically derived 95% confidence interval). The red lines shown across the scatter of the two ratios depict the regression line fit considering the variance in both ratios. The slope between the two ratios is significant for all recording sessions and is comparable across recording sessions and animals (Fig. 4*B*). The probability density estimates for the fit parameter ratio grouped by putative sleep state for all the recorded sessions indicate the three sleep states: SWS, REM/awake, and null state (Fig. 4*C*). While there is considerable overlap between the null category and the REM/awake and SWS states, it is clear that there is considerable separation between epochs when the sensorimotor cortex is characterized by rapid, low-amplitude higher–frequency activity (e.g., REM/awake) and epochs when the sensorimotor cortex is dominated by high-amplitude, slow, and low-frequency activity (e.g., SWS). This was observed in the brain states across all sessions in both subjects (TY, *d*'_SWS-REM/Awake_ = 2.51; [2.36, 2.65]_boot95%CI_; JL, *d*'_SWS-REM/Awake-SWS_ = 3.07; [2.91, 3.23]_boot95%CI_), with SWS epochs peaking at smaller parameter ratios and REM/Awake epochs peaking at larger parameter ratios. It is notable that the distribution of the fit parameter ratio for our identified SWS epochs are more tightly clustered at lower values than for the other putative categories as quantified by the coefficient of variation (TY_across sessions_: CV_SWS_ = 0.17 [0.16, 0.18]; CV_Null_ = 0.19 [0.18, 0.19]; CV_Awake/REM_ = 0.21 [0.18, 0.24]; JL_across sessions_: CV_SWS_ = 0.18 [0.17, 0.19]; CV_Null_ = 0.28 [0.26, 0.29]; CV_Awake/REM_ = 0.23 [0.20, 0.27]; bootstrap-derived 95% confidence intervals noted in brackets). Similar observations were found when the pairwise correlations were modeled as a power law model, the separation observed between the SWS and REM/awake epochs as quantified by *d*′, being more modest (Extended Data Fig. 4-2).

10.1523/ENEURO.0139-25.2025.f4-1 Figure 4-1 **Correlation by distance and parameter ratio scatters for all recording sessions. (A)** The average correlation observed at distances across the recording array for each 10 second epoch during each session reported in this study (each 10 s epoch shown in gray, mean denoted as black line). Compare with Figure 2A. **(B)** Scatter plots depict the ratio of the parameters of the fitted exponential model at each epoch during the recording to the ratio of the power in the gamma and delta bands of the LFP signal. Data is presented for all recordings reported in this study. Each 10 second epoch is color coded by the estimated sleep state (see methods). Compare to Figure 4A. Download Figure 4-1, TIF file.

10.1523/ENEURO.0139-25.2025.f4-2 Figure 4-2 **Power Law Model Description of Sleep State.** A power law model was used to describe the structure in the pairwise correlations of the LFP signal across sensorimotor cortex. **(A)** Similar to Figure 2B, the two parameters of the model were correlated. Each point is a ten second epoch during the recording session, where green points denote epochs classified as REM/awake, purple as SWS, and grey as Null epochs. **(B)** Plotting the ratio of the model parameters to the ratio of delta to gamma band power revealed a correlation with sleep state, similar to Figure 4A (see also Figure 3B). The slope from a type 2 regression (and bootstrap derived 95% confidence intervals) for each session appear in the title. **(C)** The distribution of the parameter ratio by sleep state was like that found with the exponential model, though with slightly more modest separation between SWS and REM/awake. D prime (and bootstrap derived 95% confidence intervals) appear in the title (compare with the average d-prime derived for the exponential model). Download Figure 4-2, TIF file.

## Discussion

Here, we demonstrate the reliable identification of putative SWS epochs based on spatially correlated LFP activity recorded in the sensorimotor cortex of common marmosets during nighttime sleep. We observed that the average pairwise correlation of the broadband LFP signal gradually decreased across the sensorimotor cortex when measured between any two electrodes on our array. This decrease was adequately described by a two-parameter exponential decay function—defined by an initial value and a spatial decay constant (*λ*)—fit at 10 s intervals throughout the sleep recordings. The estimated exponential decay function exhibited dynamic temporal structure, closely aligning with the well-known interplay between REM and non-REM sleep stages. These findings demonstrate that putative SWS epochs can be reliably identified using spatially correlated LFP activity across the sensorimotor cortex (Extended Data Fig. 3-2), providing further insight into the neural dynamics underlying sleep.

### Broad spatiotemporal structure in the LFP is present in the sensorimotor cortex during sleep

We found that the pairwise correlations across the entire spatial extent of our array were significantly elevated on average for the entire duration of our nighttime recordings (*ρ *∼ 0.5; Figs. 1, 2). This was true even during putative REM/Awake epochs, when neural activity has the hallmark of being desynchronized (Evarts et al., 1962; Crofts et al., 2001). The average broadband LFP signal during our sleep recordings exhibited notable spatial structure. It is generally thought that the broadband LFP signal reflects the pooled synaptic activity of neurons near the recording electrode (Pesaran, 2010), suggesting that the elevated and spatially extensive pairwise correlations may arise from a combination of external inputs, recurrent and local connectivity, and other related signals (Salelkar et al., 2018).

The elevated spatially extensive correlations observed even at large distances can be attributed to a number of factors. When we examined the LFP signal in the sleep recordings at different narrowband frequency ranges, we observed that the elevated spatially extensive correlations were largely attributed to power in the lower-frequency delta (1–4 Hz) band (Extended Data Fig. 2-2). Power in higher-frequency bands exhibited more modest correlations with the highest correlation values within 2 mm of the source electrode and at high gamma (65–150 Hz) approached zero at the furthest spatial extent of our array. At least two types of delta waves have been observed during SWS that are distinct in their cellular mechanisms: (1) delta waves driven by thalamocortical neurons and (2) delta waves generated via local cortical mechanisms (Steriade, 2003). Additionally, previous work has shown that SO/delta waves in humans demonstrate a continuum of phase differences across cortical areas that change over time, becoming more desynchronized (e.g., more local) later in sleep (Nir et al., 2011). We should note that our recordings were sampled from early in the night (∼8 P.M.–12 A.M.) of the subjects and therefore may reflect the less desynchronized more global correlation state observed in humans. These findings suggest that low-frequency LFP may play a role in mediating spatial correlations during sleep (at least within an area), potentially reflecting long-range network synchronization. In contrast, the more modest correlations across space observed here at the higher frequencies of the LFP signal may reflect local coordination of pools of spiking units near the electrode tips.

Another possible source for the elevated spatially extensive correlations observed here may result from the timescale used to estimate the pairwise correlations. Previous work analyzing the correlation of spiking activity across a planar array located in human medial temporal lobe during sleep (Peyrache et al., 2012), similarly found significant non-zero correlations across the entire extent of the array. They found that these correlations grew larger when the duration used to estimate the pairwise correlations was more extensive. In this work, we observed that reducing the timescale used to estimate the pairwise correlations increased the variance of the pairwise correlations measured over the course of the recording (Extended Data Fig. 2-1). When estimated with a 1 s timescale, the correlations across space at numerous time points fell well below the minimum correlations observed when measured with a 6 s timescale; the average correlation across the recording, however, dropped only modestly. Therefore, the timescale used to estimate pairwise correlations can only partially account for the elevated correlations observed in the broadband LFP signal. These findings demonstrate that spatially extensive correlations in neural activity can persist across large cortical areas, even during sleep, and that their magnitude is influenced by the timescale over which they are measured. This suggests that both spatial and temporal factors play a critical role in shaping large-scale coordination in neural populations.

Finally, it is also possible, that some portion of the elevated spatial correlation that we observed here is due to volume conduction of the LFP signal through the cortical tissue. While our study is primarily concerned with the relationship of brain states to spatiotemporal structure rather than the spatial extent of the LFP signal, we do acknowledge that volume conduction may play role. Overall, we observed that power in the delta band seemed to be largely responsible for the spatially extensive, high average pairwise correlations we observed during our recording, while the timescale used to estimate the pairwise correlations only modestly influence our estimates. One of the hallmarks of SWS is increases in delta band power (Diekelmann and Born, 2010; Brodt et al., 2023), which stem from some combination of the rhythmic activation of thalamocortical and local cortical circuits (Steriade, 2003). The alignment of the spatiotemporal structure observed during SWS with delta, coupled with the profound capacity for delta-related organization of population spiking, as evidenced by “up” and “down” states, suggests potential spatially organized population spiking during sleep that will have to be left for future studies of memory consolidation and reactivation.

### The spatiotemporal structure in the sensorimotor cortex observed during sleep is adequately described by an exponential decay model

We modeled this observation in the data with an exponential decay model fit at each time point. Exponential decay models have been used previously to describe the decay of neural signals across the cortex. The attenuation over distance of pairwise correlations measured between the activity of putative excitatory neurons during sleep has also been well described by an exponential decay model in the human medial temporal lobe (Peyrache et al., 2012), suggesting that the spatiotemporal structure observed here may be a more general feature of the cortex during sleep. Exponential decay has also been used to describe the stimulus-evoked spiking activity across the motor cortex of macaques due to single pulse electrical stimulation (Hao et al., 2016), further suggesting that the anatomical connections mediating functional connectivity may follow an exponential process.

Previous modeling work (Bédard et al., 2004; Lindén et al., 2011) describing the biophysical basis for the broadband LFP signal have modeled the contribution over distance of local neuronal elements to the amplitude of the LFP signal as $\frac{1}{r^{2}}$. This might suggest that the spatiotemporal structure across the cortex exhibited by the LFP signal might also be well described by a power law model. Given the ubiquitous nature of power law relationships in describing the functional properties of neural populations in the cortex (Buzsáki and Mizuseki, 2014), we also examined a power law model to describe the decay of the pairwise correlations across distance in our data (Extended Data Figs. 3-1, 3-3). We found that the power law model performed better than other models in one subject (TY) while performing comparably to a Gaussian model in the second subject (JL). In both cases, the power law fits to the data resulted in less error than that obtained with an exponential model. While the exponential model was modestly better at separating SWS and REM/awake epochs (average *d*′, power law model, 1.97; exponential model, 2.79; Extended Data Fig. 4-2), both models were adequate in identifying the spatiotemporal structure prevalent in the different epochs identified here during sleep across the sensorimotor cortex.

Our findings also confirm previous work (Destexhe et al., 1999) showing that spatial structure of pairwise LFP correlations can be described with an exponential model. However, prior studies fit the exponential decay function to these correlations using linear regression without accounting for the skewed, non-normal sampling distribution of the Pearson's correlation coefficient or its nonstable variance. To address these limitations, we applied Fisher's *z*-transform (Fisher, 1915) to the correlation values. This transformation unbounded the correlation values from the constrained [−1,1] domain, improving the stability of variance and leading to a better fit of the exponential decay function to the transformed data [compare Fig. 2*B* with Destexhe et al. (1999); Fig. 1*A*,*C*].

Applying the Fisher transform also affected the evaluation of spatial structure at short distances. In untransformed data, correlations near zero distance are expected to be 1 (perfect correlation), but the transformation removed this upper bound, revealing finer details in the decay of spatial correlation. Notably, we found that time points with elevated spatial correlations were primarily characterized by smaller spatial decay constants and larger initial values in our two-parameter exponential decay model, rather than by a larger spatial decay constant, as previously reported (Destexhe et al., 1999). While our results align with prior findings that certain time points exhibit more spatially extensive correlations during sleep, they also refine this interpretation. Rather than a flatter decay profile, we observed that increased correlations were more pronounced at shorter distances within the sensorimotor cortex during these periods (Fig. 2*B*). This suggests a more localized enhancement of functional connectivity rather than a uniform expansion across space. Together, these findings provide a more focused and complementary perspective on the spatiotemporal structure of functional connectivity in the marmoset sensorimotor cortex during sleep.

### Limitations of study

We did observe differences on average in the decay of the pairwise correlation across distance between subjects (Fig. 2*A*; Extended Data Fig. 3-1). These differences may reflect larger structural differences across M1 and PMd due to array placement. While the arrays in both subjects targeted the forelimb representation and covered the full anterior to posterior extent of M1, they also covered portions of 3a/3b in the somatosensory cortex posteriorly and PMd anteriorly to varying extents between the two subjects (Fig. 1*A*). The array in subject TY covered larger portions of 3a/3b than the array in JL, while approximately half of JL's array was estimated to cover PMd. Studies of anatomical projections to forelimb M1 in the marmoset reveal pervasive connectivity with 3a/3b and PMd (Burish et al., 2008; Burman et al., 2008, 2014), suggesting extensive coordination between these areas in natural behavior. Electrical stimulation in the posterior aspect of PMd (or Area 6d in marmosets) near M1 has been reported to produce movements largely in the shoulder, elbow, and trunk while being largely unresponsive in more anterior areas (Burish et al., 2008). Intracranial microsimulation mapping of JL's array also largely indicated activity of the shoulder and trunk in the anterior half of the array, with some of the most anterior electrodes being unresponsive. In subject TY, there were clear wrist, hand, and forearm responses in the anterior portions of the array, suggesting that the anterior extent of TY's array was more centrally placed in M1 than JL's array. Functionally, while there is clear evidence that both M1 and PMd can encode movement parameters and have directional tuning in macaques (Caminiti et al., 1991; Ben-Shaul et al., 2003), there is also clear evidence that neurons in PMd also participate in movement planning (Riehle and Requin, 1993). These structural and functional qualities of M1 versus PMd may contribute to the differences we observed in the spatial correlation structure between our two subjects. Overall, however, the variance in the pairwise correlation values across the recordings largely overlap in both subjects: the average correlation varies from *ρ* ∼ 0.8 at the smallest distances, gradually decreasing over space for ∼2 mm before generally spreading to a greater or lesser extent around *ρ* ∼ 0.5.

Additionally, we lacked electromyography and oculography during sleep recordings and thus cannot make finer distinctions within sleep stages. In the current study, we relied on the well-documented observation of increases in delta power during SWS and corresponding decreases in gamma power during REM/awake activity (Crofts et al., 2001; Stickgold, 2005), to classify sleep stages based solely on the LFP activity using methods similar to those that have been reported in studies with rats (Kim et al., 2019; Silversmith et al., 2020). Additionally, we did observe clear alternating epochs of putative awake/REM and SWS in our recordings (Fig. 3*A*), indicative of the classic sleep cycle and suggesting that we were observing sleep qualitatively similar to what has been reported previously in marmosets (Crofts et al., 2001; Bukhtiyarova et al., 2022). Our third putative sleep state (the “null” category) was defined as 10 s epochs not well represented by the LFP dynamics that classically define the other two states. While it is not possible to determine what physiological state is explicitly represented by the “null” category with only ratios of delta and gamma band power, this null category likely corresponds to states dominated by lighter stages of NREM sleep. Additionally, because each point is the average power in a 10 s epoch, the null category could also include transitions between putative SWS and REM states or transitions between putative SWS and awake states, brief interruptions in either SWS or REM/awake states, or noise in the signal contributed by either the subject or sleeping companion movement. This interpretation is supported by the quantification of head movement from video obtained during a sleep recording (Extended Data Fig. 3-3). This analysis found that the amount of head movement during null epochs was intermediate between the amount of movement during SWS and REM/awake epochs. Furthermore, by placing the “null” epochs in a different category, our sleep classification ensures that only epochs that are dominated by LFP dynamics consistent with the well-accepted definitions of deep NREM sleep states were considered in our analysis.

We examined the spatiotemporal structure of the broadband LFP signal in the forelimb sensorimotor cortex during natural sleep in the common marmoset. We observed that sleep is characterized by a pervasive and broad spatiotemporal coherence in the LFP signal that closely matched the temporal structure of REM and non-REM sleep. In addition to confirming previous work (Destexhe et al., 1999), we also extended those results by showing that pairwise correlations become more pronounced at shorter distances during epochs we classified as SWS. Additionally, we generalized previous results with a spatially extensive, multiregional view of the forelimb sensorimotor cortex by taking advantage of the marmoset's lissencephalic cortex and the use of chronic planar recording arrays. Finally, our results show that the average spatiotemporal correlation across space can be used to recover epochs characterized by LFP power dynamics that are consistent with SWS stages of sleep and suggest a broader more general correlation with sleep architecture, setting the stage for investigations of the spatial organization of sleep dependent sensorimotor memory consolidation by identifying putative SWS periods during sleep.

## Synthesis

Reviewing Editor: Silvia Pagliardini, University of Alberta Decisions are customarily a result of the Reviewing Editor and the peer reviewers coming together and discussing their recommendations until a consensus is reached. When revisions are invited, a fact-based synthesis statement explaining their decision and outlining what is needed to prepare a revision will be listed below. The following reviewer(s) agreed to reveal their identity: Agustín Solano.

Reviewer#1

In this work, the authors explore the spatial correlation in the LFP and use it to identify slow wave sleep. The technical execution of the work seems reasonable, but its scientific impact is unclear. The use of electrical stimulation to verify the location of the recording electrodes is a valuable control, yet this work suffers from several issues.

My main concern is that the results may be due to volume conduction rather than a biological correlation induced by sleep. Additionally, the authors introduce and discuss memory and motor learning, but these topics are not relevant to the very technical (potentially only biophysical) result presented in the manuscript. Also, most of the results would benefit from a more clear description, and to be framed in the context of recent literature.

Can the authors exclude that the results are due to volume conduction (e.g. using Laplacian transform or Bipolar referencing? Or the envelope of the spiking activity or the related gamma modulation associated with slow waves?)? Particularly, the slower frequency content of slow waves facilitates their volumetric spread, and may lead to results analogous to the reported ones without any larger biological synchronization. Furthermore, the exponential spatial decay reported may be in line with such hypothesis. This hypothesis may be further corroborated by Figure S2. Excluding that the results are driven by volume conduction is critical as, if that's the case, this would invalidate most of the interpretation proposed by the authors about neuronal populations and sources of LFP activity.

The authors refer to thalamocortical neurons generating slow waves. Yet, they overlook a large body of literature (started by Nir, Neuron, 2011) demonstrating that slow waves are frequently a local event.

The authors introduce and discuss the topic of memory and motor learning. Sleep has several functions, and memory and motor learning represent a small niche that is not relevant to the present manuscript. If the authors want to overview the effects of sleep (that in my opinion goes beyond the purpose and results of this paper), a more exhaustive revision of literature would be required.

The manuscript focuses on some statistical and analytical features of the data whose biological meaning is unclear. Clarifying their biological meaning would greatly increase the impact of the manuscript for a neuroscience audience.

Minors

The authors state that "marmosets are diurnal sleepers like humans". This sentence is unclear to me, since it suggests that marmosets and humans sleep during the day

What is the meaning of the null category?

In the introduction, the authors refer to NREM/REM sleep as a feature of mammals and birds. Yet, NREM/REM sleep has been reported in reptiles about a decade ago (Shein-Idelson, Science, 2016)

Figure 1:

The schematic at the bottom left of Figure 1A is unclear. What is it showing?

The authors show a green trace, but the green electrode is the reference electrode(and thus no signal should be recorded from it). How is this possible?

Panel B is unclear. What are the authors pointing with the black brackets?

Figure 3:

The authors report values of REM/awake and SWS. It would be helpful to report REM and awake separated. Also, What is the meaning of the "null" epochs?

The authors report frequent signal dropout. A rough quantification of these signals would be useful to the reader (e.g. average duration and frequency).

The figure titles seem to be partially disconnected form what is shown in the respective figures. Also, the authors refer several times to "spatiotemporal structure" when only the spatial structure is actually investigated.

Typo: line 160 "was"

The use of the weighting in the average is unclear. Was it used somehow differently from the weighting in the model?

Unclear: "performing a linear regression on a function of the Pearson correlation.".

To describe "micro" units, please use "μ" instead of "u".

The authors mention the slow sleep power, using a frequency band distinct from delta. How is it used? Is it relevant for the present manuscript?

Some panels on the right side of Figure 1A seems to be misaligned and cover the axes labels. Also, some of the labels are missing.

The abbreviation MSE is not introduced.

Reviewer #2

This study presents a novel and generally methodologically sound approach to identifying slow-wave sleep (SWS) in marmosets by analyzing the spatiotemporal correlations of local field potentials (LFP) over the sensorimotor cortex. The main finding is that SWS epochs are reliably characterized by distinct LFP spatial correlation patterns, specifically exhibiting higher initial correlation values and lower spatial decay constants compared to other REM/Awake stages. A strong linear relationship and significant positive correlation were found between the ratio of the fit parameters (λ/A) and the ratio of gamma/delta narrowband power. Notably, the distribution of the fit parameter ratio for SWS was more tightly clustered at lower values compared to other sleep stages. The study concludes that spatially correlated LFP activity can reliably identify SWS epochs in the sensorimotor cortex, offering new insights into sleep-related neural dynamics and suggesting potential applications for studying sleep-dependent memory consolidation.

Comments

-The study presents interesting findings regarding the relationship between spatially correlated LFP activity and SWS identification in the marmoset sensorimotor cortex. The importance of SWS for memory consolidation and the suitability of the marmoset model for studying sleep-dependent processes relevant to humans are well-established. The objective to use spatial LFP correlations as a method to identify SWS is also clearly stated. While the Introduction provides valuable context, the specific rationale for pursuing the identification of SWS using spatial LFP correlations (as opposed to traditional methods like power spectral analysis, which you also use for validation) could be explicitly reinforced at the beginning of the final paragraph of the Introduction (lines 110-118). Clarifying why looking at the spatiotemporal coherence of LFP would help memory consolidation research would strengthen the introduction. Please also include a clear working hypothesis to further understand the rationale of the study. In addition, it is important to clearly state that the current study focuses solely on characterizing the neural signature of SWS and does not provide direct evidence for its role in memory consolidation in these marmosets.

-You chose to use an exponential model for the correlation decay with distance. Please provide a clearer justification for this choice. You mention exponential decay has been used previously (e.g. Destexhe et al.), which is a valid reason, but given the prevalence of power law relationships in neural data (e.g., neural avalanches, 1/f noise, network structure; Lindén et al., 2011; Buzsáki &Mizuseki, 2014), could a power law model also be a plausible alternative for your correlation by distance data? Have you formally compared the fits between exponential and power law models using metrics like AIC or BIC to definitively determine the best mathematical explanation for your data? If not, discussing the potential relevance of power law models in this context would be valuable, even if the exponential model was ultimately chosen based on fit or prior literature.

-The Gaussian Mixture Model identified three clusters: SWS, REM/Awake, and a "null state" where delta/SO and gamma band power were approximately equivalent. You note that this "null state" is abundant and shows considerable overlap with the other categories in the probability density functions (Figure 4C). While the absence of EMG/EOG is a limitation you acknowledge, could you discuss in more detail what this "null" state might physiologically represent (e.g., transition periods, lighter stages of NREM sleep, brief periods of quiet wakefulness not captured by the "REM/Awake" criteria)? Have you explored alternative clustering methods or classifiers that might provide better separation or a more physiologically meaningful categorization? Even without EMG/EOG, behavioral video data exists, could it be used to provide some validation or context for these "null" periods?

-In lines 329-330, please clarify what you mean by "most 'active' (e.g. REM/awake) and the 'least active' (e.g. SWS) brain states". Their relation to the regression between the ratios of spatial parameters and power ratios might not be immediately obvious.

-Expand in the discussion on how your specific results regarding the spatial correlation patterns during SWS might mechanistically support the study of sensorimotor memory consolidation. What specific future investigations are enabled by being able to identify SWS via these spatial patterns?

-I have a minor disagreement with the phrasing in the last sentence of the discussion: "Finally, our results show that the average spatial-temporal correlation across space can be used to recover traditional SWS stages of sleep...". While your spatial correlation parameters are highly correlated with the traditional power-based SWS classification, you haven't explicitly demonstrated that these spatial parameters alone are sufficient to "recover" or classify SWS stages with a high degree of accuracy. Have you performed a classification or clustering analysis using only the spatial correlation parameters to see how well it predicts the power-based SWS labels? If so, presenting the results of such an analysis would directly support the claim that these parameters can be used to recover SWS stages. If not, the wording might be softened slightly.

-At lines 286 and 324, please correct the wording to explicitly state what "significant" means in the context of the statistical test performed.

-Please clarify what the values reported in the parentheses represent in lines 308-311.

-For correlations across distance (Figure 2A) and the scatter plots of ratios (Figure 4A), it would be highly beneficial to include the figures corresponding to all recording sessions using the 10-second windows in the supplementary materials. This would allow readers to further explore the variability across sessions and between animals, strengthening the presentation of your data.

Minor Comments

-Define NHP (Non-Human Primate) at line 109.

-I suggest enhancing the labels in Figure 3A to improve clarity. Positioning the labels for both Delta/Gamma power and the REM/Awake-SWS classification centrally between the two panels might make it clearer that they apply to both plots shown in that figure.
