# Supplementary figures and images for: Spatially Extensive LFP Correlations Identify Slow-Wave Sleep in Marmoset Sensorimotor Cortex
Source: eNeuro. 2025 Nov 6;12(11):ENEURO.0139-25.2025. doi: 10.1523/ENEURO.0139-25.2025 (PMC12604550; doi:10.1523/ENEURO.0139-25.2025)

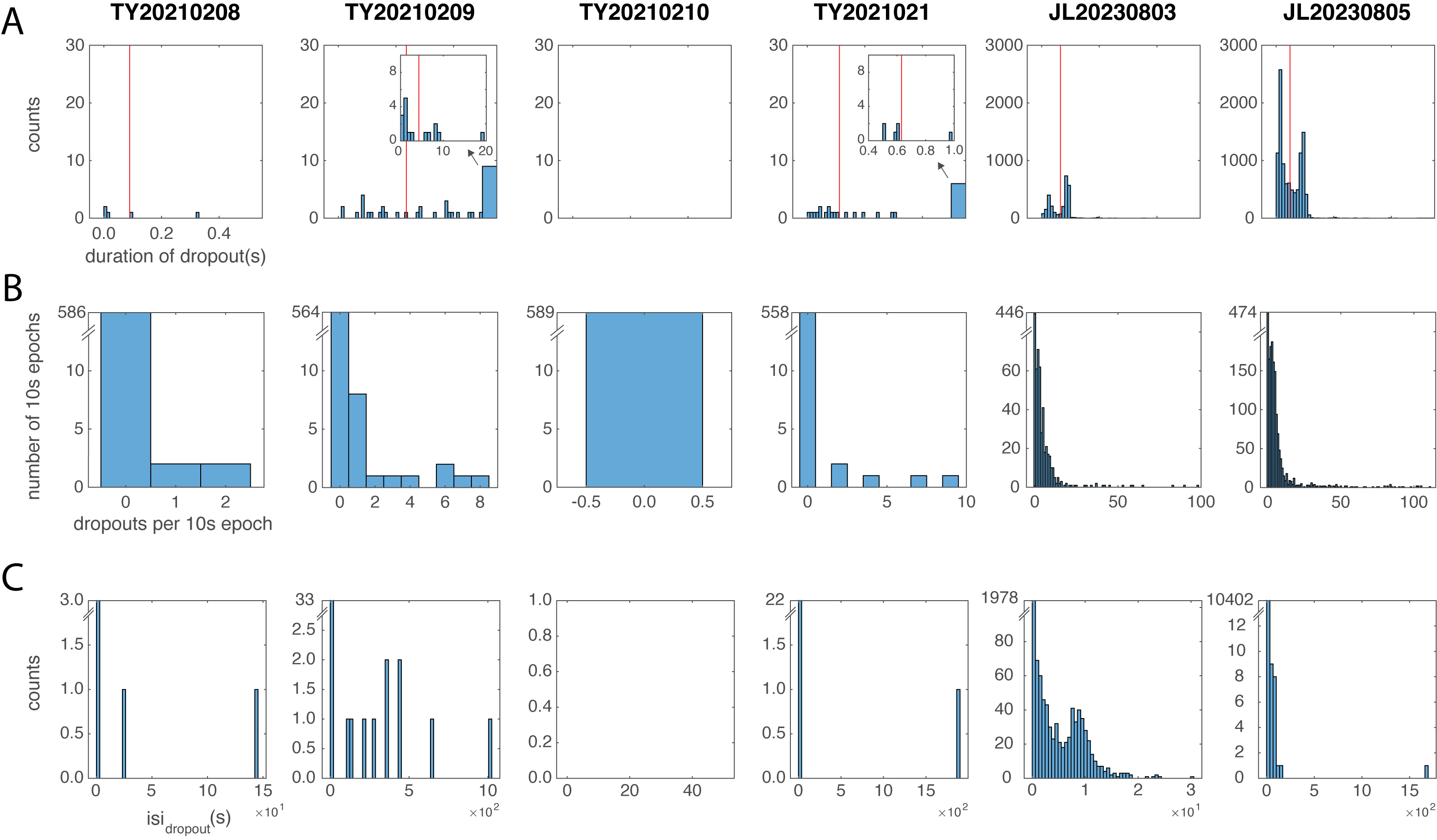

Supplement: Figure 1-1 — Characterization of Wireless Signal Dropout During Sleep Recordings. (A) Duration of wireless signal dropouts: Histograms depict the duration of dropouts observed during all recording sessions. Fewer instances of signal dropout were observed in subject TY’s recordings, which were generally fewer and of longer duration than observed with subject JL. All x-ranges identical in main axes of (A). Insets show the distribution for the longer duration dropouts in subject TY’s recordings. Red lines depict the mean of the distribution. (B) Frequency of Dropouts: Histograms depict the number of 10 second epochs containing the number of dropouts listed on the x-axis. Note that there were no dropouts observed in session TY20210210, so all the epochs had zero dropouts. (C) Intersample interval for the dropout distribution in each recording. In all sessions, most of the dropouts happened in quick succession (the bin closest to zero is the largest). Download Figure 1-1, TIF file. [file eneuro-12-ENEURO.0139-25.2025-s001.tif]

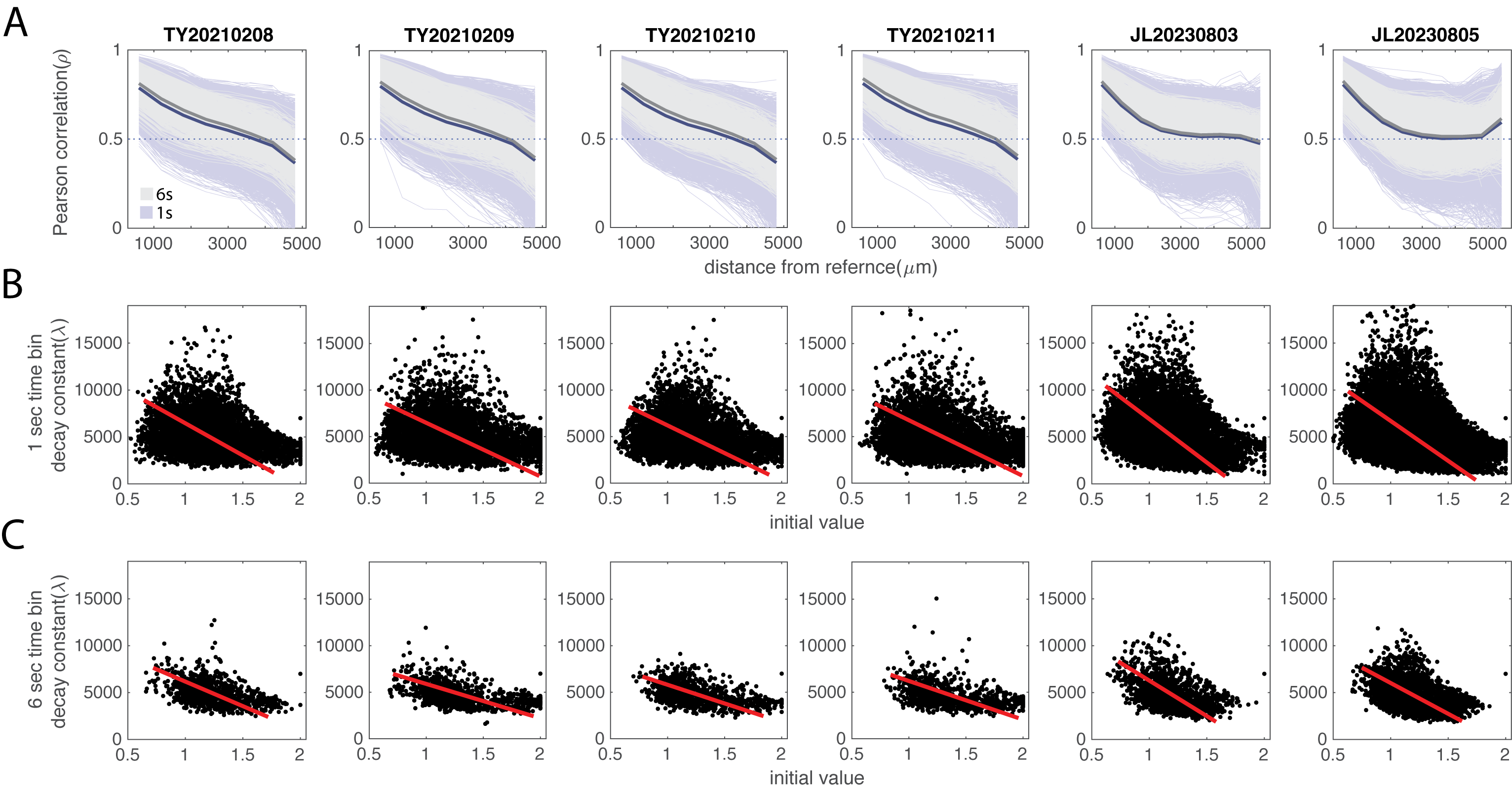

Supplement: Figure 2-1 — Pairwise distance correlations estimated using different timescales. (A) The average, across electrode, pairwise correlation as a function of distance across the array is plotted for each time point during the four recording sessions in TY and two recording sessions in JL. The lilac traces depict the results when a timescale of 1 second was used to estimate the pairwise correlations, while the lighter gray represents the same data estimated on a 6 second timescale. The thicker line of similar hue represents the mean. Shorter timescales produce a larger variance in the distribution of the correlation over space, and result in lower correlations on average: the mean of the distance functions for the 1 second timescale (heavy grey lines in A) is lower than it is for the 6 second timescale (heavy blue lines in A). (B,C). Scatter plots for the exponential model parameters fit to the data in each recording session shown in (A) for the 1 second and 6 second time scales are showing the same qualitative trend as the broadband data: the spatial decay constant (λ) estimated from the data is anticorrelated with fitted initial value parameter. Download Figure 2-1, TIF file. [file eneuro-12-ENEURO.0139-25.2025-s002.tif]

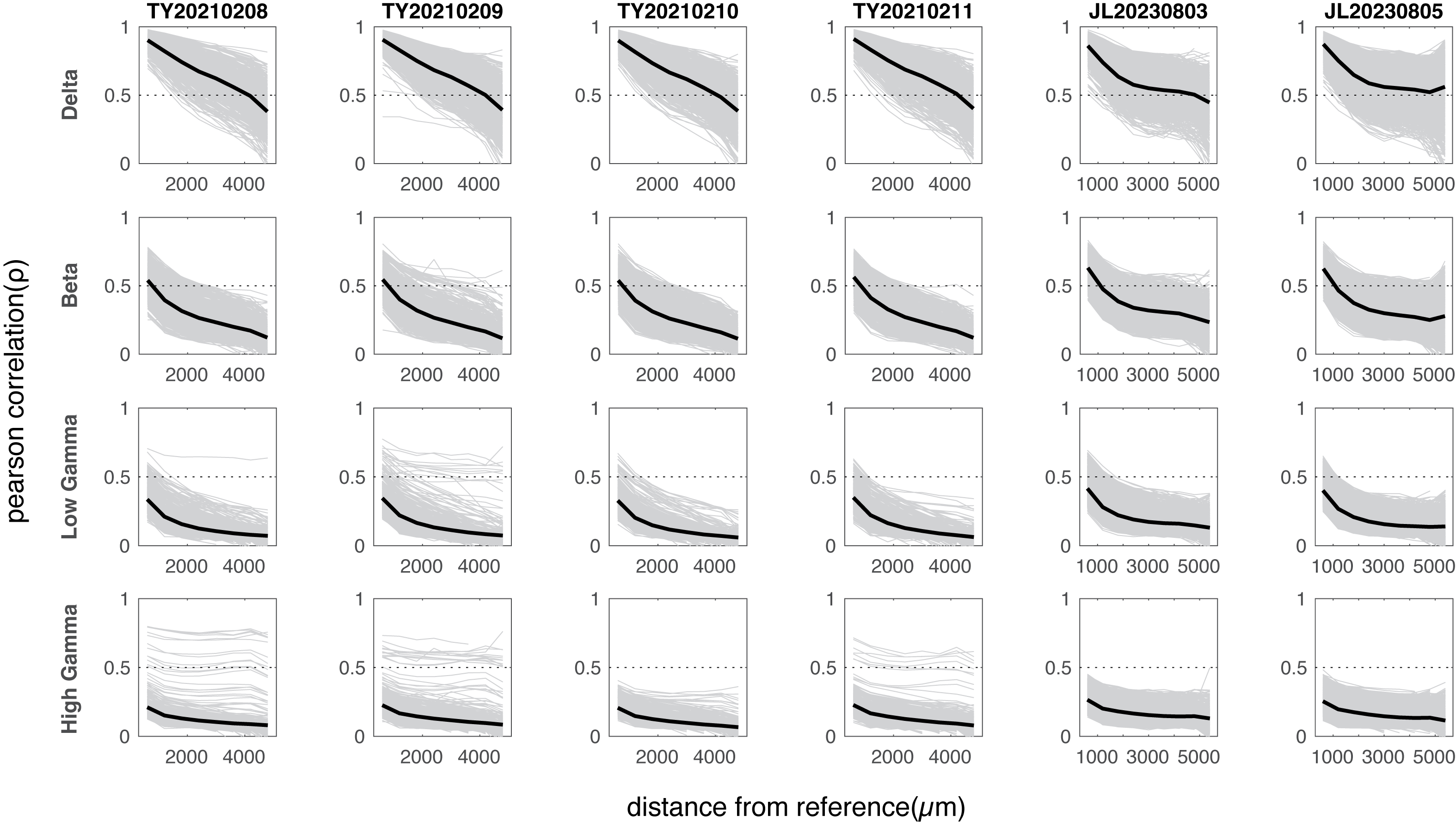

Supplement: Figure 2-2 — Average pairwise correlation over distance in narrowband frequencies. The narrowband power in the delta (1-4 Hz), beta (16-24 Hz), low gamma (25-55 Hz), and high gamma (65-140 Hz) frequencies were analytically derived from the broadband LFP signal obtained during nighttime sleep recordings in two marmoset subjects (TY and JL). Each plot depicts the average (across electrodes) pairwise correlation over the spatial extent of an array located in the forelimb representation of sensorimotor cortex (gray lines). The average over time is depicted as the black line in each plot. Spatially extensive high correlations are observed dominantly in the delta band across both subjects and all sessions. The peak correlation, in addition to the overall correlation, decreases in higher frequency bands. Download Figure 2-2, TIF file. [file eneuro-12-ENEURO.0139-25.2025-s003.tif]

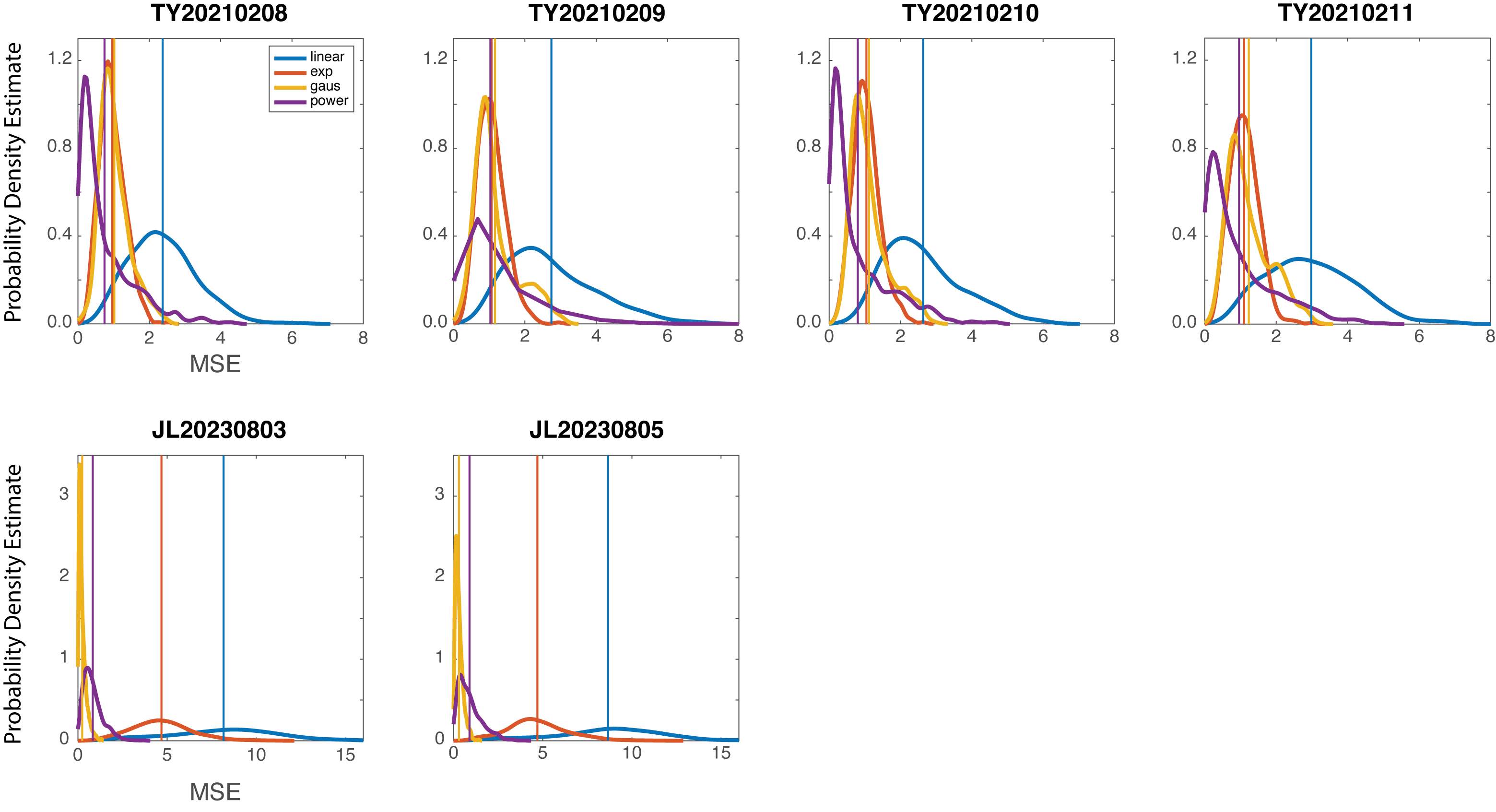

Supplement: Figure 3-1 — Fit error for models of the average pairwise correlation as a function of distance. Linear, exponential, gaussian and power law models were fit to the average pairwise correlation across space in both subjects. The smoothed probability density estimates for each distribution of the mean squared error (MSE) is plotted for each model. Density estimates were carried out on the empirically observed distribution of the residuals fit for each model using Matlab’s ksdensity function (Mathworks, Natick, MA). A vertical line notes the mean of each distribution. The distribution of the MSE for the power law decay model tended to peak at lower values in subject TY, but comparable to the Gaussian model in subject JL. In subject TY, the distribution for exponential model is comparable to the performance of the Gaussian model across all the recording sessions. In subject JL, the error from the exponential model is broad and distributed at a higher mean than the Gaussian model. Linear models provide the highest error in both subjects. Download Figure 3-1, TIF file. [file eneuro-12-ENEURO.0139-25.2025-s004.tif]

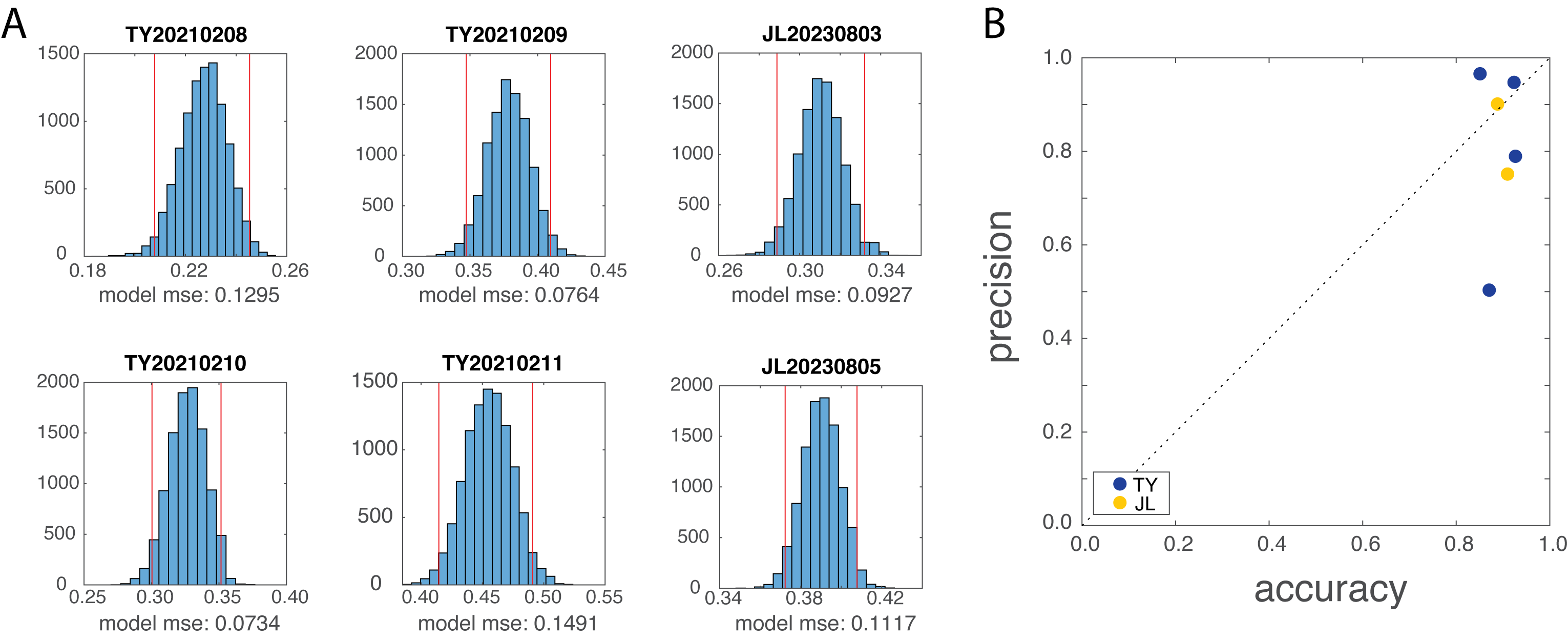

Supplement: Figure 3-2 — Simple Threshold Model Significantly Predicts Putative SWS Epochs. We tested a model generated by thresholding the initial value parameter fitted to each time point in a session in predicting SWS epochs on each recorded session. The fitted parameter was also smoothed in time. The threshold and smoothing value were obtained by minimizing the resulting error of the prediction on all the other sessions in a leave-one-out training paradigm. (A) The error distributions from shuffling the SWS labels show that the model prediction error (listed below each distribution) for each session was well below the 95% confidence interval of the shuffle distribution. (B) Estimates of accuracy (the proportion of hits and correct rejections) and precision (the proportion of hits to the sum of hits and false alarms) for each session. One session had a relatively low precision as shown in the plot. This session was also the one that had the least number of SWS epochs. Overall, this very simple model did reasonably well in predicting which epochs had LFP dynamics consistent with SWS. Similar results were found using the same threshold/smoothing model with the decay constant or the ratio of decay constant to initial value as the parameter to threshold (not shown). Download Figure 3-2, TIF file. [file eneuro-12-ENEURO.0139-25.2025-s005.tif]

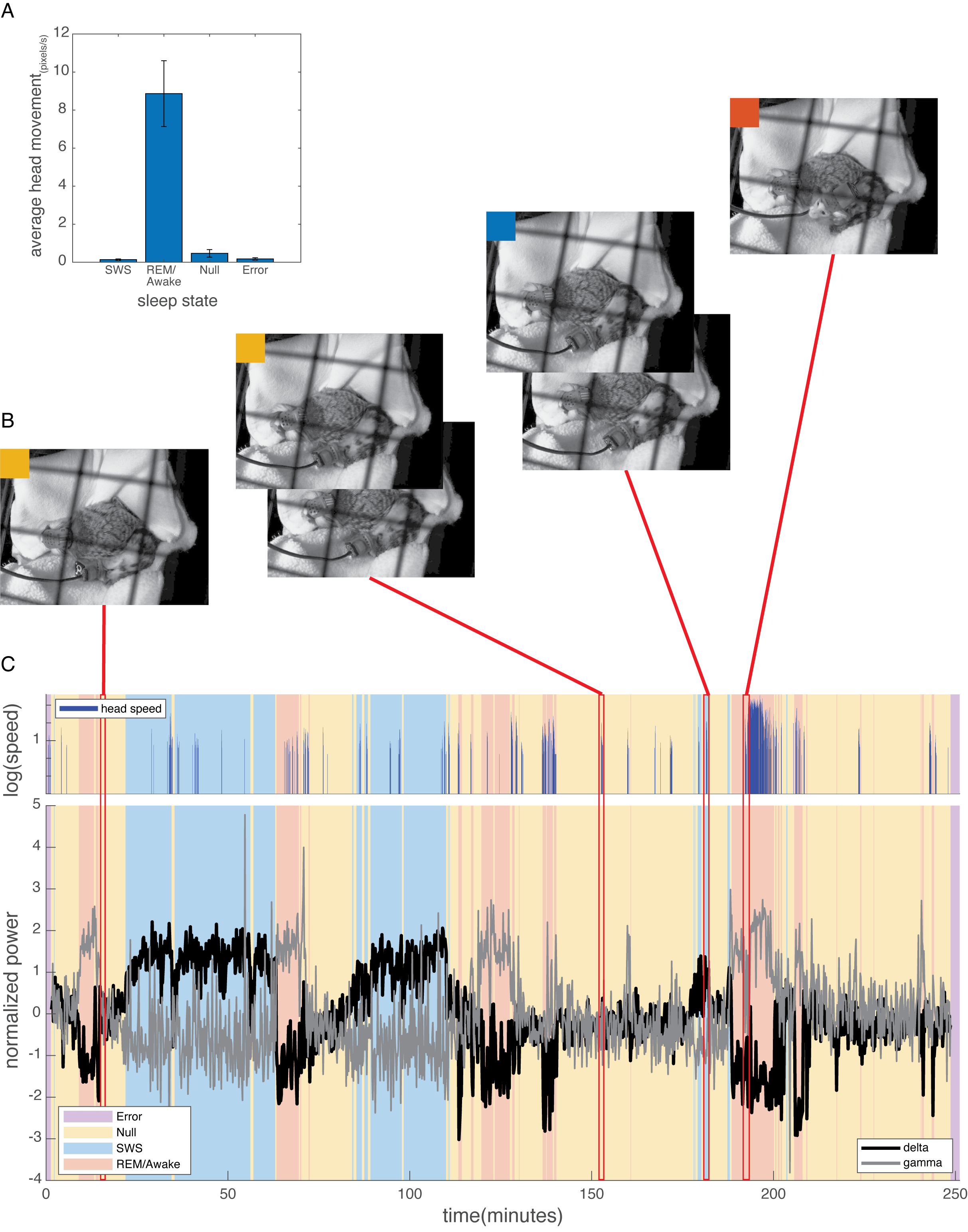

Supplement: Figure 3-3 — Video Analysis of Movement During Sleep. Video of sleep session was used for validating of sleep scoring. Note the neural recording headstage and charging cable indicating the subject of recording. Subject movement was quantified by tracking the movement of the subject’s head in each videoframe using the progressive tracking (Mielke et al., 2020). The x-y pixel-wise position of a point on the recording headstage was converted to head speed over the entire course of the recording, and sleep staging was conducted using the same 3 state (SWS, REM/Awake, and Null) model reported in the paper. (A) The average speed in each state over the entire recording shows that most movement occurred during REM/Awake, with a non-significant trend of movement in the Null state being larger than that during SWS. (B) Video frames depicting movement during example time points displayed in (C). The sleep state is shown on the colored background, with log (head speed) plotted on the upper plot in blue and delta (0.1-4 Hz) and gamma (30-60 Hz) power plotted in black and gray on the bottom plot. A significant amount of movement is observed in the last REM/Awake epoch, where the animal is clearly shown awake. The Null epochs contain both periods of movement and no movement, as depicted in the first two video example frames, and likely represent transition states between REM and NREM states, lighter NREM states, and noise. Download Figure 3-3, TIF file. [file eneuro-12-ENEURO.0139-25.2025-s006.tif]

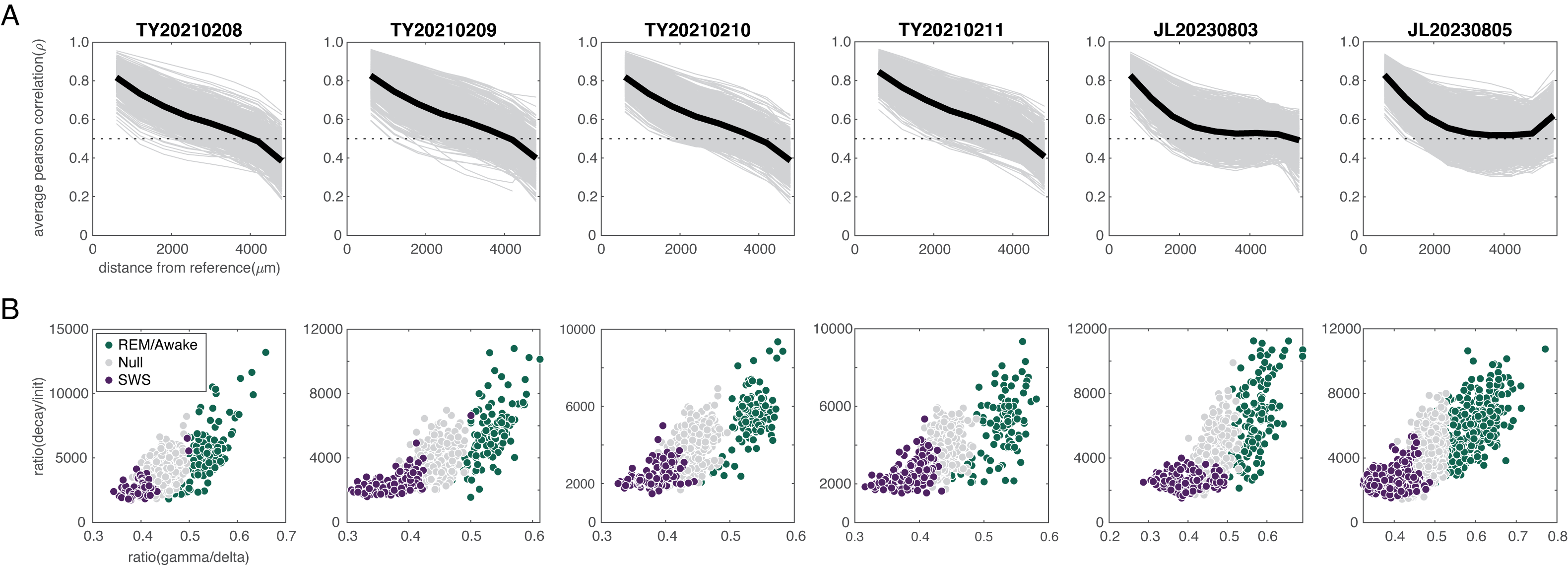

Supplement: Figure 4-1 — Correlation by distance and parameter ratio scatters for all recording sessions. (A) The average correlation observed at distances across the recording array for each 10 second epoch during each session reported in this study (each 10 s epoch shown in gray, mean denoted as black line). Compare with Figure 2A. (B) Scatter plots depict the ratio of the parameters of the fitted exponential model at each epoch during the recording to the ratio of the power in the gamma and delta bands of the LFP signal. Data is presented for all recordings reported in this study. Each 10 second epoch is color coded by the estimated sleep state (see methods). Compare to Figure 4A. Download Figure 4-1, TIF file. [file eneuro-12-ENEURO.0139-25.2025-s007.tif]

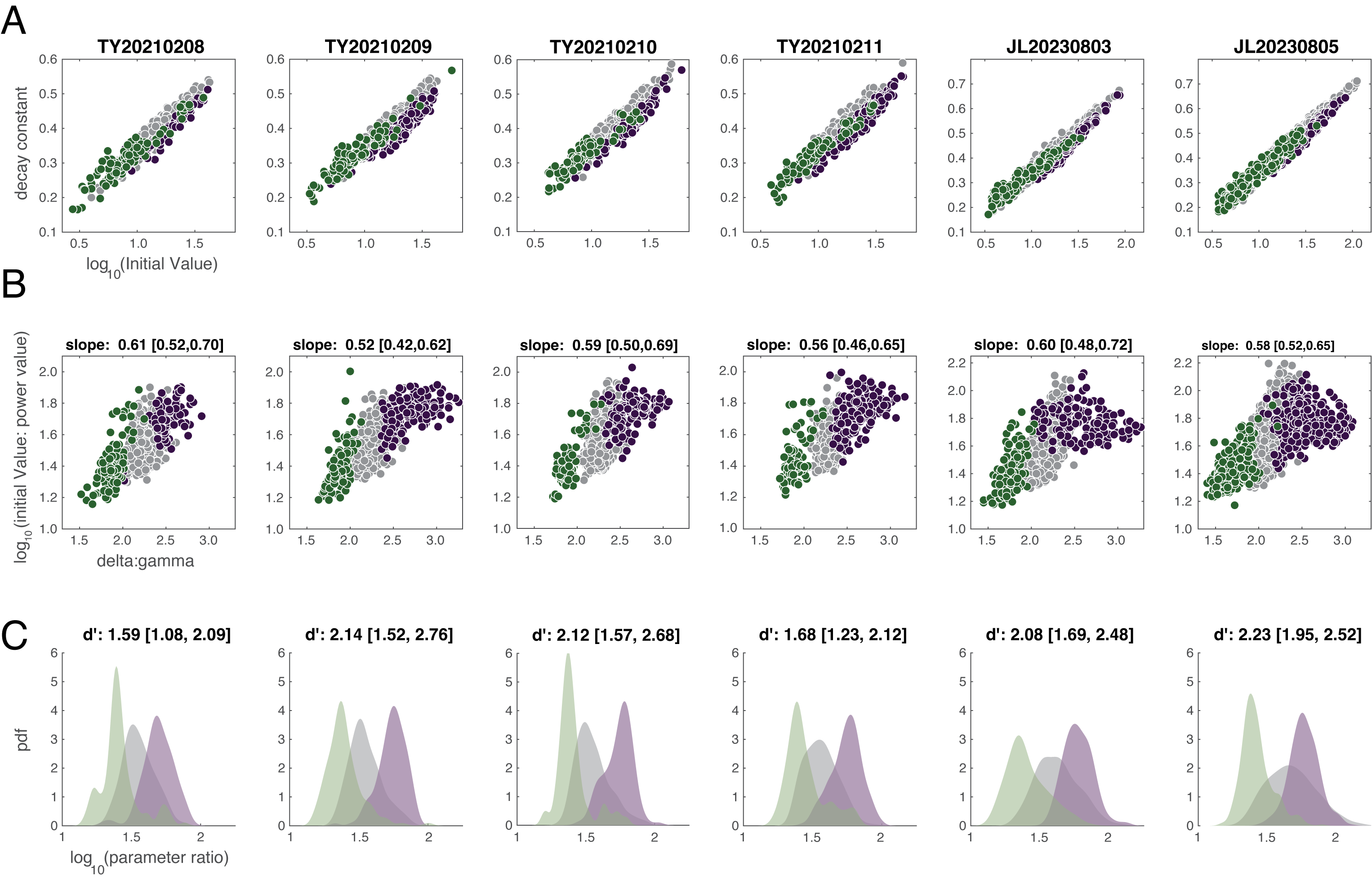

Supplement: Figure 4-2 — Power Law Model Description of Sleep State. A power law model was used to describe the structure in the pairwise correlations of the LFP signal across sensorimotor cortex. (A) Similar to Figure 2B, the two parameters of the model were correlated. Each point is a ten second epoch during the recording session, where green points denote epochs classified as REM/awake, purple as SWS, and grey as Null epochs. (B) Plotting the ratio of the model parameters to the ratio of delta to gamma band power revealed a correlation with sleep state, similar to Figure 4A (see also Figure 3B). The slope from a type 2 regression (and bootstrap derived 95% confidence intervals) for each session appear in the title. (C) The distribution of the parameter ratio by sleep state was like that found with the exponential model, though with slightly more modest separation between SWS and REM/awake. D prime (and bootstrap derived 95% confidence intervals) appear in the title (compare with the average d-prime derived for the exponential model). Download Figure 4-2, TIF file. [file eneuro-12-ENEURO.0139-25.2025-s008.tif]
